# Systems biology of the structural proteome

**DOI:** 10.1186/s12918-016-0271-6

**Published:** 2016-03-11

**Authors:** Elizabeth Brunk, Nathan Mih, Jonathan Monk, Zhen Zhang, Edward J. O’Brien, Spencer E. Bliven, Ke Chen, Roger L. Chang, Philip E. Bourne, Bernhard O. Palsson

**Affiliations:** Department of Bioengineering, University of California, La Jolla, San Diego, CA 92093 USA; Joint BioEnergy Institute, Emeryville, CA 94608 USA; Bioinformatics and Systems Biology Program, University of California, La Jolla, San Diego, CA 92093 USA; National Center for Biotechnology Information, National Library of Medicine, National Institutes of Health, Bethesda, MD 20894 USA; Department of Systems Biology, Harvard Medical School, Boston, MA 02115 USA; Office of the Director, National Institutes of Health, Bethesda, MD 20894 USA

## Abstract

**Background:**

The success of genome-scale models (GEMs) can be attributed to the high-quality, bottom-up reconstructions of metabolic, protein synthesis, and transcriptional regulatory networks on an organism-specific basis. Such reconstructions are biochemically, genetically, and genomically structured knowledge bases that can be converted into a mathematical format to enable a myriad of computational biological studies. In recent years, genome-scale reconstructions have been extended to include protein structural information, which has opened up new vistas in systems biology research and empowered applications in structural systems biology and systems pharmacology.

**Results:**

Here, we present the generation, application, and dissemination of genome-scale models with protein structures (GEM-PRO) for *Escherichia coli* and *Thermotoga maritima*. We show the utility of integrating molecular scale analyses with systems biology approaches by discussing several comparative analyses on the temperature dependence of growth, the distribution of protein fold families, substrate specificity, and characteristic features of whole cell proteomes. Finally, to aid in the grand challenge of big data to knowledge, we provide several explicit tutorials of how protein-related information can be linked to genome-scale models in a public GitHub repository (https://github.com/SBRG/GEMPro/tree/master/GEMPro_recon/).

**Conclusions:**

Translating genome-scale, protein-related information to structured data in the format of a GEM provides a direct mapping of gene to gene-product to protein structure to biochemical reaction to network states to phenotypic function. Integration of molecular-level details of individual proteins, such as their physical, chemical, and structural properties, further expands the description of biochemical network-level properties, and can ultimately influence how to model and predict whole cell phenotypes as well as perform comparative systems biology approaches to study differences between organisms. GEM-PRO offers insight into the physical embodiment of an organism’s genotype, and its use in this comparative framework enables exploration of adaptive strategies for these organisms, opening the door to many new lines of research. With these provided tools, tutorials, and background, the reader will be in a position to run GEM-PRO for their own purposes.

**Electronic supplementary material:**

The online version of this article (doi:10.1186/s12918-016-0271-6) contains supplementary material, which is available to authorized users.

## Background

The success of genome-scale modeling can be attributed to high-quality, bottom-up reconstructions of metabolic, protein synthesis, and transcriptional regulatory networks on an organism-specific basis [1–4]. Such network reconstructions are biochemically, genetically, and genomically (BiGG) structured knowledge bases [5] that can be used for discovery purposes (such as model-driven discovery of unidentified metabolic reactions [6], studies of evolutionary processes [7], and analysis of biological network properties), as well as practical applications (such as metabolic engineering, prediction of cellular phenotypes [8], and interspecies similarities and differences). Others have explored host/pathogen interactions [9], cocultures and microbial communities [10–13], ecology [14], and chemotaxis [15]. Numerous recent developments have broadened the predictive scope of genome-scale models by incorporating other sources of biological data, such as protein structural data, into reconstructions [7, 16, 17].

**Fig. 1:**
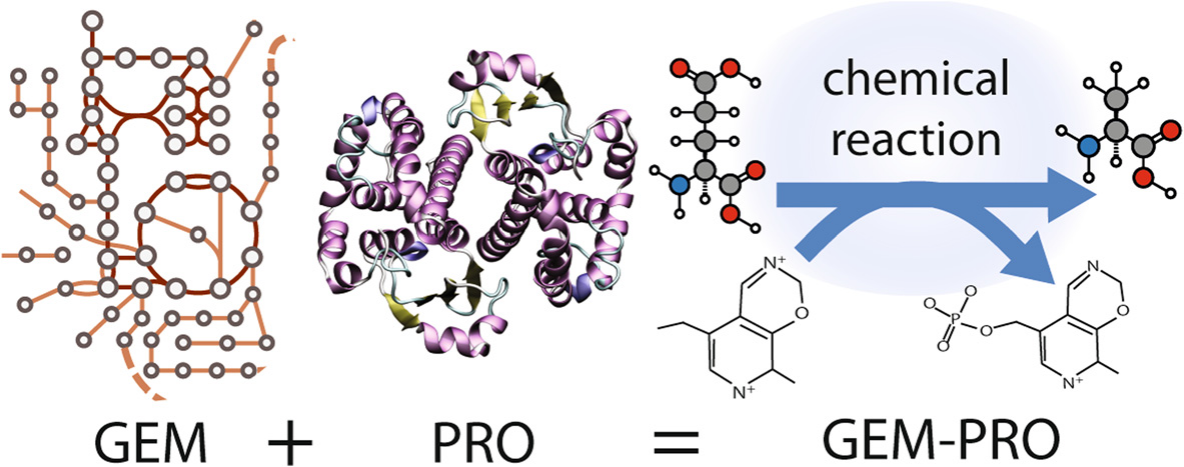
Structural systems biology emerges from the integration of networks and structural biology. Genome-scale models incorporate multi-omic data and large-scale curation from databases such as KEGG and UniProt. Molecular-level analyses enable atomic-level characterizations of secondary structure, substrate binding, and comparisons of similar catalytic sites among proteins in the metabolic network

The complementarity of molecular-level and systems-level data types has led to the integration of protein structurally-derived data into genome-scale models. Using genome-scale models of metabolism (GEMs), we link metabolic enzyme activities to characteristics of observed phenotypes, whereas using structural biology, we link molecular interaction details (e.g., protein-ligand binding) to the activities of enzymes. The genome-scale models with protein structures (GEM-PRO) framework, therefore, gives a direct mapping of gene to transcript, to protein structure, to biochemical reaction, to network states, and finally to phenotype (Fig. 1). Understanding the structural properties of proteins as well as their respective ligand binding events (e.g., metabolite, drug or oncometabolite) enables the characterization of molecular-level events that trigger changes in states of an entire network. Such a multi-scale approach acts as bridge between systems biology and structural biology, two scientific disciplines that, when combined, become the emerging field of structural systems biology [18–22]. This union has brought about exciting advances, which would have otherwise been out of reach: the evolution of fold families in metabolism [7], identification of causal off target actions of drugs [16], identification of protein-protein interactions [23, 24], and determination of causal mutations for disease susceptibility [24, 25].

In recent years, the number of publicly available biological macromolecule structures has grown to more than 110,000 entries, and continues to increase yearly by roughly 10 % [26]. The increasing availability of protein structural data brings about a number of implications for GEM-PRO models. First, to keep pace with the deluge of protein data coming from experiments, there is a developing need for pipelines that use systematic mapping and quality assurance processes to read, filter, and process all newly deposited structures, ultimately managing all relevant data in an easy-to-use knowledgebase. Second, increasingly accessible protein structural data enhances the predictive scope of systems biology research; the more description we have of the biological components involved in complex systems, the more we can understand cellular processes that span a wide range of biological, chemical, and structural detail. Expanding these models would allow for the progressive description from a 1 to a 2- to a 3-D view of biology. Finally, to aid in the dissemination and further development of these resources, growing datasets and pipelines should be developed together with *in silico* tools that increase data accessibility and training.

Here, we address each of the above implications and demonstrate how linking protein structural data to GEMs enables the generation, dissemination, and application of GEM-PRO for studying two contemporary organisms, *T. maritima* and *E. coli*. For the generation and updating of GEM-PRO, we present a novel pipeline that systematically maps genes in a metabolic model to their respective high-quality structural data. We present four novel applications areas which demonstrate the utility of modeling at the intersection of systems and structural biology: (i) metabolic protein specificity; (ii) the relationship between protein complex stoichiometry and in vivo protein abundance; (iii) the diversity of bacterial proteomes; (iv) protein properties of growth rate-limiting reactions at high temperatures. Finally, for dissemination and training purposes, we distribute the GEM-PRO knowledgebase together with tutorials, which explicitly describe how GEM-PRO can address the following questions: (i) How are protein fold families distributed over metabolism? (ii) How does temperature, and hence protein instability, determine growth rate?

## Results and discussion

### Generation and updating of GEM-PRO using a systematic pipeline

As with metabolic network reconstructions [1], structural proteome reconstructions require constant curation and updating to incorporate newly deposited experimental protein structures. For example, over the course of two years, the number of available experimentally determined protein structures for *E. coli* has increased substantially (since 2013, 356 additional experimental *E. coli* protein structures can be linked to genes in the metabolic network model, *i*JO1366 [27]) and the structural coverage of genes in the model has increased by 10 % (133 genes). In this section, we describe the construction of a quality assessment pipeline which enables newly deposited crystallographic or NMR structures to be searched, assessed, and managed within a structured k-base. In total, 2 person-hours are required by this workflow, once all homology models have been constructed for proteins without available crystallographic structures. Time and computational requirements for homology modeling are discussed in the I-TASSER pipeline [28]. The workflow discussed here can be carried out with no specific hardware requirements, and software requirements are outlined within the tutorial notebooks.

#### Coverage of protein structures in metabolism

**Fig. 2:**
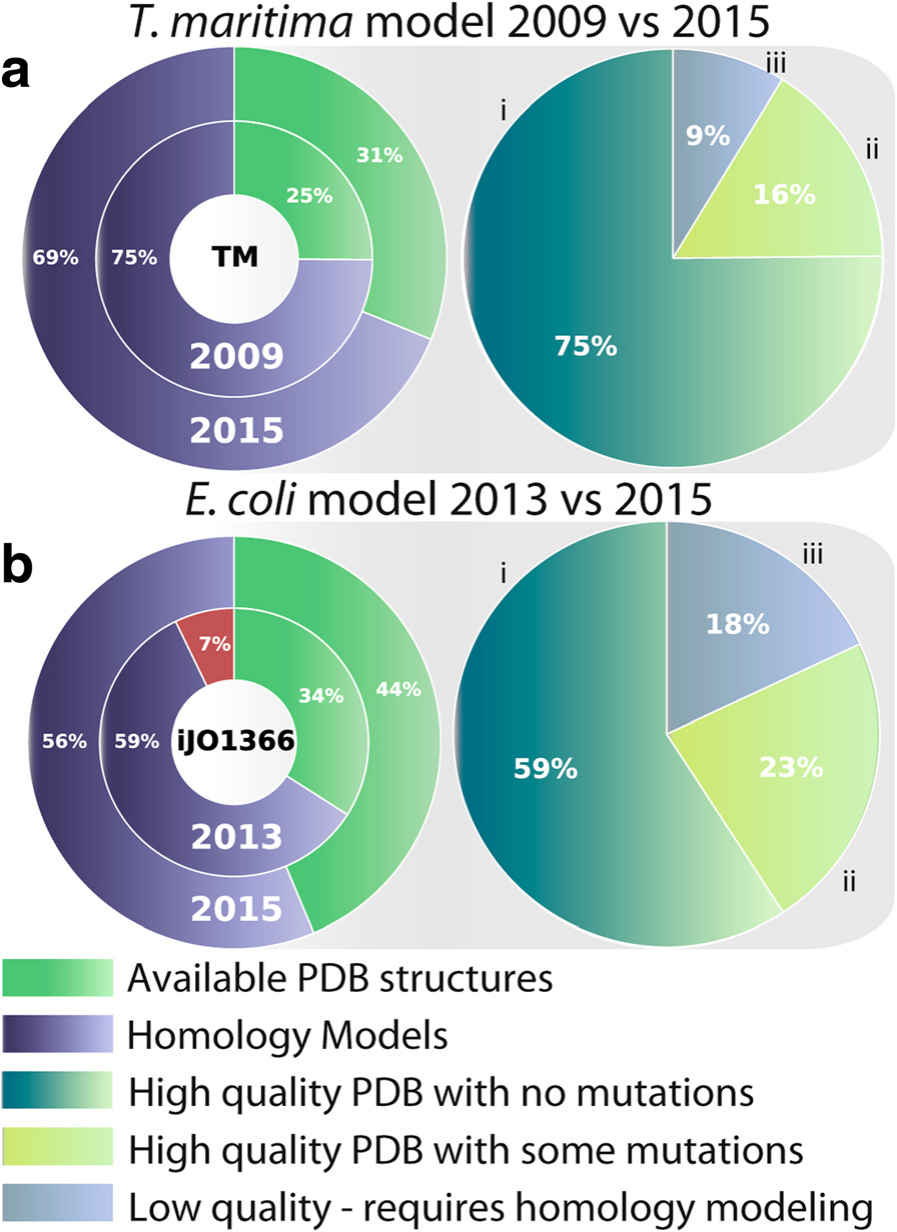
**a** The new GEM-PRO model for *T. maritima* (TM). Displayed in the pie chart on the left is the coverage of genes by a PDB structure or homology model, and a comparison of those structures available in 2009 versus 2015. In the pie chart on the right, the available PDB structures are further classified into three groups based on the overall quality of the structure: (i) high quality structures that have no mutations in the interior of the protein (112 genes involved in 210 reactions; in *teal*); (ii) high quality structures that have some mutations and require minimal modification to revert back to wild-type sequence (24 genes involved in 49 reactions; in *light green*) and low quality structures (13 genes involved in 20 reactions; *blue*) that may have large gaps of unresolved sections of the protein or a large number of mutations at the interior of the protein and require further homology modeling (in *light blue*). Determining the quality of a PDB is explained in detail in the section entitled *Quality control and quality assessment of all structures.* The same quality assessment evaluations were carried out for *E. coli* in (**b**)

**Fig. 3:**
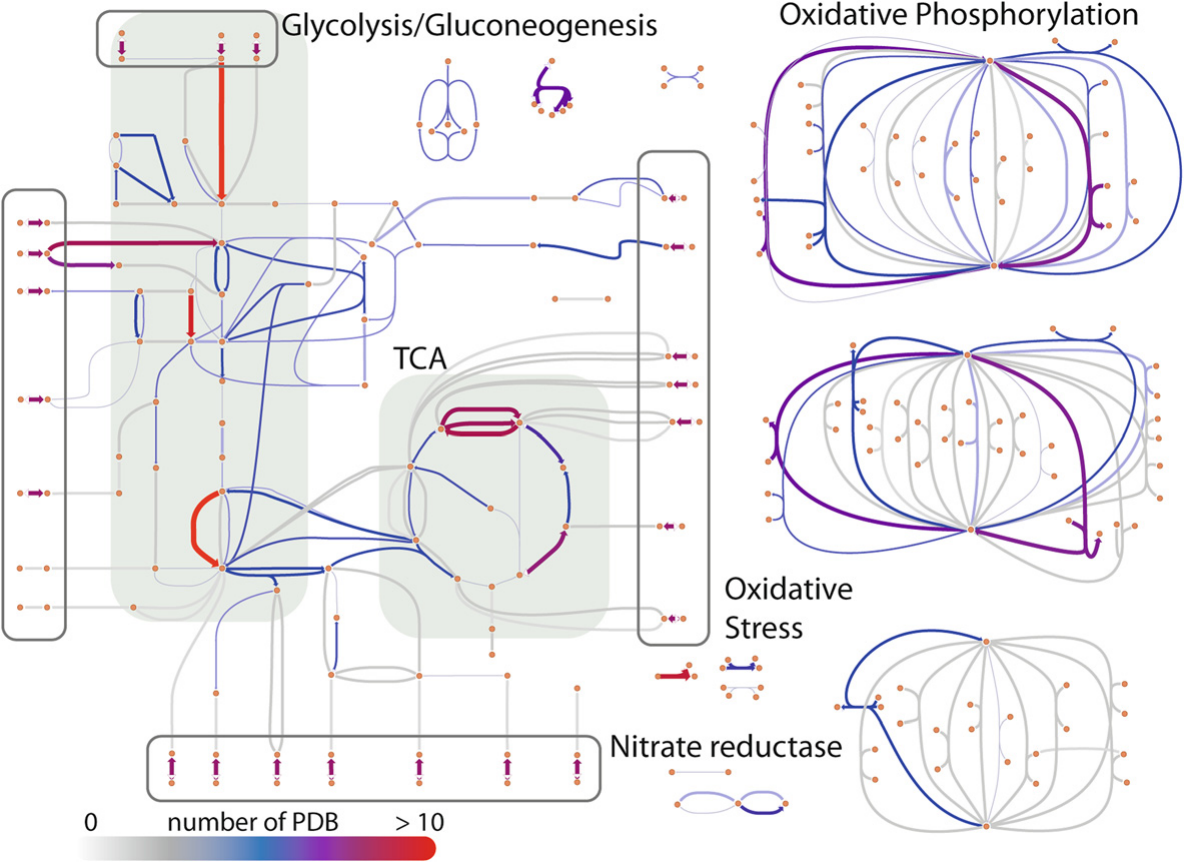
All available PDB structures mapped to the network of *E. coli* metabolism (*i*JO1366 model [27]). The heat map indicates an increase in the number of available experimental protein structures that map to a given reaction in the pathway (*grey* to *blue* to *red* transitions represents 0 to more than 10 PDB structures). Subsystems such as glycolysis and TCA are highlighted by the colored grey squares and transporters by transparent rectangles with grey borders. The largest increase in coverage in subsystems involved in alanine and aspartate metabolism, glycolysis and gluconeogenesis, folate metabolism, cysteine metabolism, the citric acid cycle, arginine and proline metabolism, tRNA charging, and nitrogen metabolism

We find that the coverage of all experimental (X-ray crystallography and NMR) protein structures (PDB) for genes in *T. maritima* and *E. coli* is between 30–45 %, which is 6–10 % higher compared to the original GEM-PRO reconstructions (Fig. 2). The updated GEM-PROs for *T. maritima* (*i*BM478-GP) and *E. coli* (*i*BM1366-GP) include 336 and 3425 PDB structures, respectively, an additional 5–10 % of newly deposited protein structures compared to the original versions (see inner versus outer nested pie chart in Fig. 2). Of the newly deposited protein structures, the majority are linked to subsystems in metabolism with a higher coverage of protein structures compared to others (e.g., alanine and aspartate metabolism, see Fig. 3 and Additional file 1: Figures S3 and S4).

As shown in Fig. 2, nearly 56–69 % of genes in the GEMs cannot be mapped to available experimental protein structural information. To a large extent, the 3D structure of a protein can be estimated from homology modeling, which predicts structure based on experimental templates of proteins that are homologous in sequence to the protein of interest. Here, we selected the I-TASSER (iterative threading assembly refinement) suite of programs [29, 30], which has been the highest ranking program for automated protein structure prediction for the the past two CASP experiments [30–33]. Mapping the *E. coli* model to available I-TASSER homology models [24, 34, 35], we find that the coverage is nearly complete for its metabolic proteome (1343 genes have available template-based homology models and 23 have ab initio models [34]). For *T. maritima*, we have performed homology modeling using the I-TASSER protocol to generate models for a total of 333 genes lacking experimental protein structure information. We find that the updated GEM-PRO models make use of over 100 recently deposited (and higher quality) experimental structures compared to the previous models (see Additional file 1).

#### Quality of experimental and homology-based structures

**Fig. 4:**
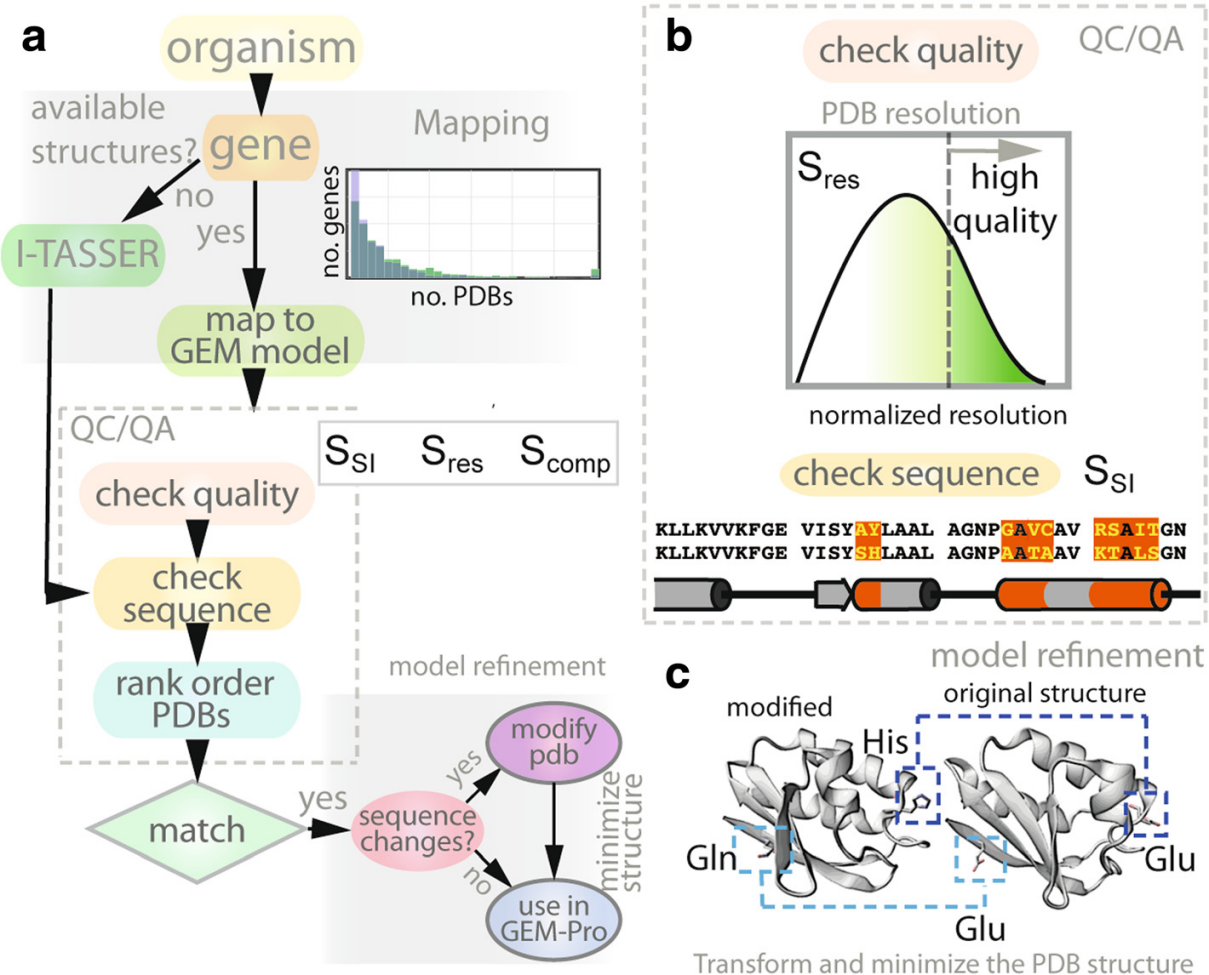
Workflow for generating simulation-ready models of all proteins in metabolism. **a** The first stage involves mapping the genes of the organism to available crystallographic and NMR protein structures, found in the Protein Data Bank (PDB). The second stage performs homology modeling for genes without available structures. The third stage performs ranking and filtering of structures and homology models for each gene based on set selection criteria (e.g., S_SI_, S_res_ and S_comp_). These criteria refer to a scoring metric that ranks a PDB structure based on sequence identity (S_SI_), resolution (S_res_), or homology model based on the similarity in secondary structure composition (S_comp_) compared to the structure. As shown in **b**, evaluation of the sequence identity between the protein structure sequence and that of the wild-type sequence and PDB resolution (in Å) allows filtering of low-quality structures. In the final stage, all high quality PDB files that require minimal modification (e.g., reversion of the sequence to match that of the wild-type) are further refined, as depicted in (**c**)

In many cases, experimental protein structures may contain unresolved fragments of the protein or mutations in the sequence (often as artifacts or the result of a crystallization protocol or due to natural disorder). Small variations in sequence can have large-scale effects on the structure and function of proteins. Thus, we perform a rigorous assessment of the quality of all structural data for each model organism. To determine which experimental structures require further modeling (e.g., group iii proteins, displayed in Fig. 2a and b) or minimal modification (e.g., group ii proteins, displayed in Fig. 2a and b), we devised a scoring metric that ranks each PDB structure based on a set of criteria: the maximum coverage of the wild-type amino acid sequence, PDB resolution, and minimal number of missing or unresolved parts of the structure (see Fig. 4b and Additional file 1 for more details).

In the previous *E. coli* GEM-PRO, 43 % of all proteins contained unresolved fragments. After carrying out the QC/QA pipeline, we correct for all cases and provide 100 % complete (gap-less) and sequence identical structures of proteins. To further assess the quality of protein structures in the updated GEM-PRO, we have evaluated all structures using PROCHECK [36], which assesses the stereochemical quality of a protein structure, and PSQS, based on statistical potentials of the mean force between residue pairs and between solvent and residue [37]. While the average quality scores for all protein structures in the updated versions of GEM-PRO are similar to those of previous versions, the completeness of all structural models in the updated GEM-PROs substantially enhances the quality of the structures in the model and their capacity for future applications.

#### Structural and sequence refinement of structures

**Fig. 5:**
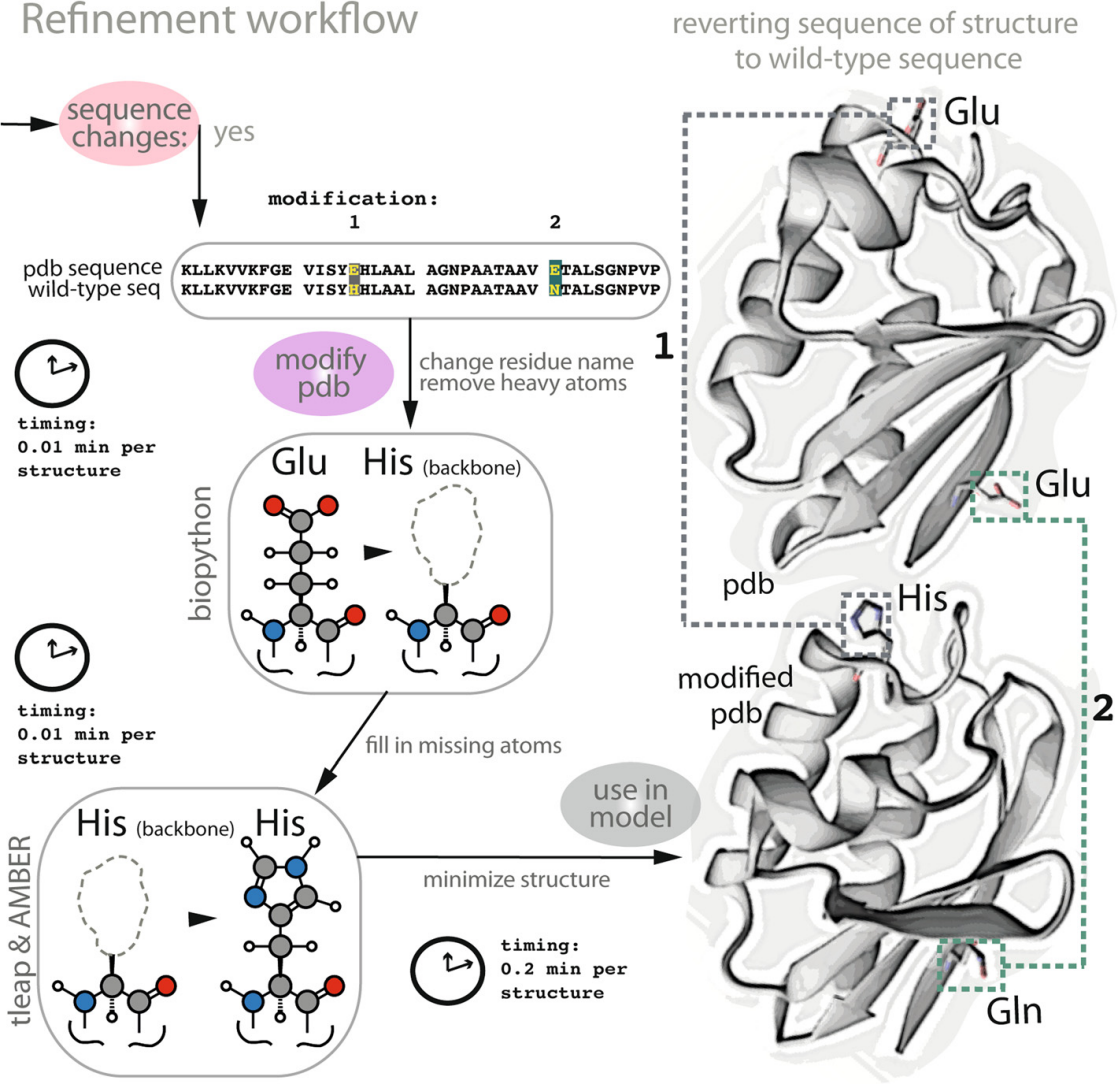
This workflow demonstrates the final stage of refinement for PDB structures, performed to replace atomic coordinates of atoms in a mutated residue with atomic coordinates corresponding to the wild-type residue. Using a combination of Biopython modules and the AMBER suite of programs, each PDB structure is modified and the final structure is minimized. For example, an original crystal structure and its wild-type sequence differ by two residues (Glu115His and Glu131Gln). The modified structure is reverted back to the original wild-type sequence in three stages: (i) all atoms in the R-group of the target amino acid (except for the peptide backbone atoms) are stripped from the file; (ii) new atoms with their respective 3D atomic coordinates are placed in the “empty” amino acid ‘site’ (e.g., the R-group atoms of Glu); (iii) the modified structure undergoes energy minimization using a steepest descent algorithm to relieve any bad contacts (i.e., steric hindrance) that may be caused by the addition of new atoms

The final step in the workflow (Fig. 4c) carries out minimal sequence modifications of nearly perfect, high-quality experimental structures (e.g., group ii proteins, displayed in Fig. 2a and b). Modifications of this set of structures are mainly needed to fix: (i) a minimal number of single-residue mutations (i.e., not more than two sequential mutations); or (ii) a minimal number of deletions or missing residues in the interior of the protein. This final step enables one of the most considerable improvements in the updated GEM-PRO framework, providing a complete set of minimally modified experimental structures that have 100 % sequence identity to wild-type sequence. Using our PDB refinement pipeline (Fig. 5), we find that 16 % (24/136) and 23 % (136/490) of experimental protein structures in the GEM-PRO of *T. maritima* and *E. coli*, respectively, require minimal modifications to revert the PDB sequence to the wild-type sequence. See Table 1 for details on average sequence identity and completeness.

#### Final outcome of mapping protein structures to genome-scale data

**Fig. 6:**
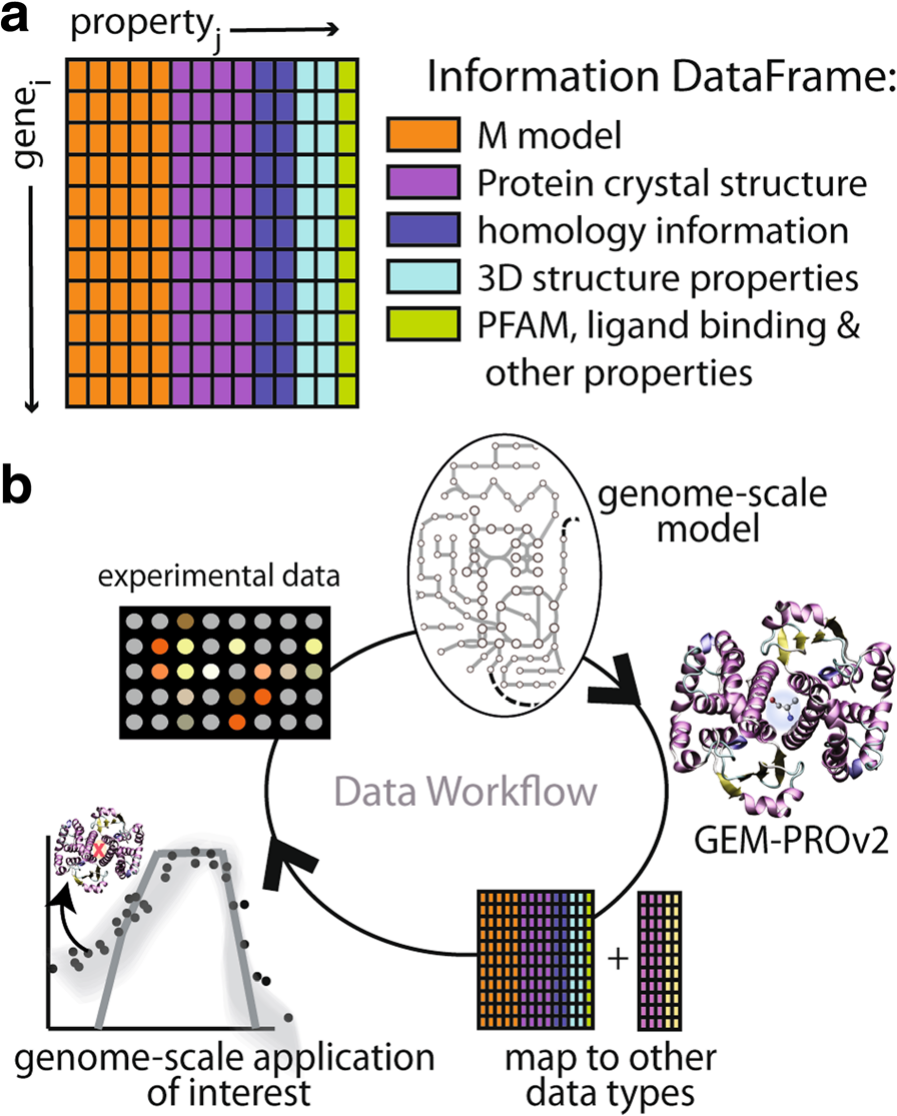
**a** The master GEM-PRO data frame which stores various protein-related properties for a specified organism. **b** A proposed data workflow, in which a genome-scale model is integrated with protein structural information, thus forming a GEM-PRO which can then be mapped to other data types, such as melting point temperatures, and can subsequently be applied to genome-scale applications, such as predicting growth rate of *E. coli* at different temperatures. Finally, these *in silico* predictions are compared to experiments for validation

The overall coverage and quality of the selected experimental and homology-based structures for each organism is detailed in Table 2. This database increases the scope and capacity of genome-scale models when applied within a model and data-driven workflow, As shown in Fig. 6a, the combination of protein data (e.g., melting temperature) and a genome-scale model of metabolism can be used to predict the effect of temperature on the growth rate of a model organism. These *in silico* findings can then be tested with experiments to provide input into the next round of this iterative workflow (Supplementary IPython notebook, titled “Temperature_Dependent_Growth_ Prediction.ipynb”).

**Table 1:** Quality statistics of all available protein structures in GEM-PRO models

Property	*T. maritima*	*E. coli*
Mean sequence identity	92.1 ± 15.8 %	91.8 ± 16.4 %
Mean completeness	92.3 ± 15.5 %	91.9 ± 16.1 %
Mean resolution	2.2 ± 0.5 Å	2.3 ± 1.0 Å

Mean sequence identity, completeness, and resolution refers to the average of the three metrics over all experimental protein structures in GEM-PRO. The standard deviation is given for each metric. Mean sequence identity refers to exact amino acid matches between sequence and structure, while mean completeness disregards exact matches

**Table 2:** Quality statistics of GEM-PRO models

Model	PDB coverage^a^	Homology model coverage^b^	PDB quality score^c^	Homology model quality (TM-score)^d^
*T. maritima*	136/478	342	0.82 (0.86)	0.79
*E. coli*	490/1366	1366	0.77 (0.95)	0.82

^a^Number of total genes with PDB structures (includes minimally modified) after QC/QA; ^b^Number of total genes with homology models. Note that there may be overlap between PDB and homology model coverage; ^c^Mean quality score of PDB structures in the GEM-PRO model for all available PDB structures. In parentheses are the subset of “best representative structures” for all metabolic gene (as ranked by the QC/QA pipeline), scaled (0, 1] where 0 is low quality and 1 is the highest quality; ^d^Mean quality score of the homology models taken from the I-TASSER TM-score metric, is the range [0,1] with a value >0.5 implying correct topology of the model [28]

### Modeling at the intersection of systems and structural biology

Once a GEM-PRO database has been constructed, it can be queried and used in conjunction with experimental data and genome-scale modeling approaches to understand the nature of the underlying biology. Here, we present four novel case studies which demonstrate how properties derived from the structures of proteins determine systems-level behavior.

#### Characterizing the degree of diversity in substrate specificity of metabolic proteins

Evaluating protein structural properties together with their binding capacities provides insight into structure-function relationships of isozymes and proteins that catalyze similar reactions. We are interested in using GEM-PRO to formulate hypotheses about which proteins are most likely to act promiscuously on substrates other than their native one (i.e., substrate ambiguity). Assessing the degree of substrate ambiguity with EC numbers has been explored through evaluation of fourth digit of the enzyme commission number (e.g., 2.6.1.**X**) [38]. Here, we take a different approach, we apply GEM-PRO to evaluate the degree of diversity in the substrates/ligands bound to crystallized proteins within various EC families.

**Fig. 7:**
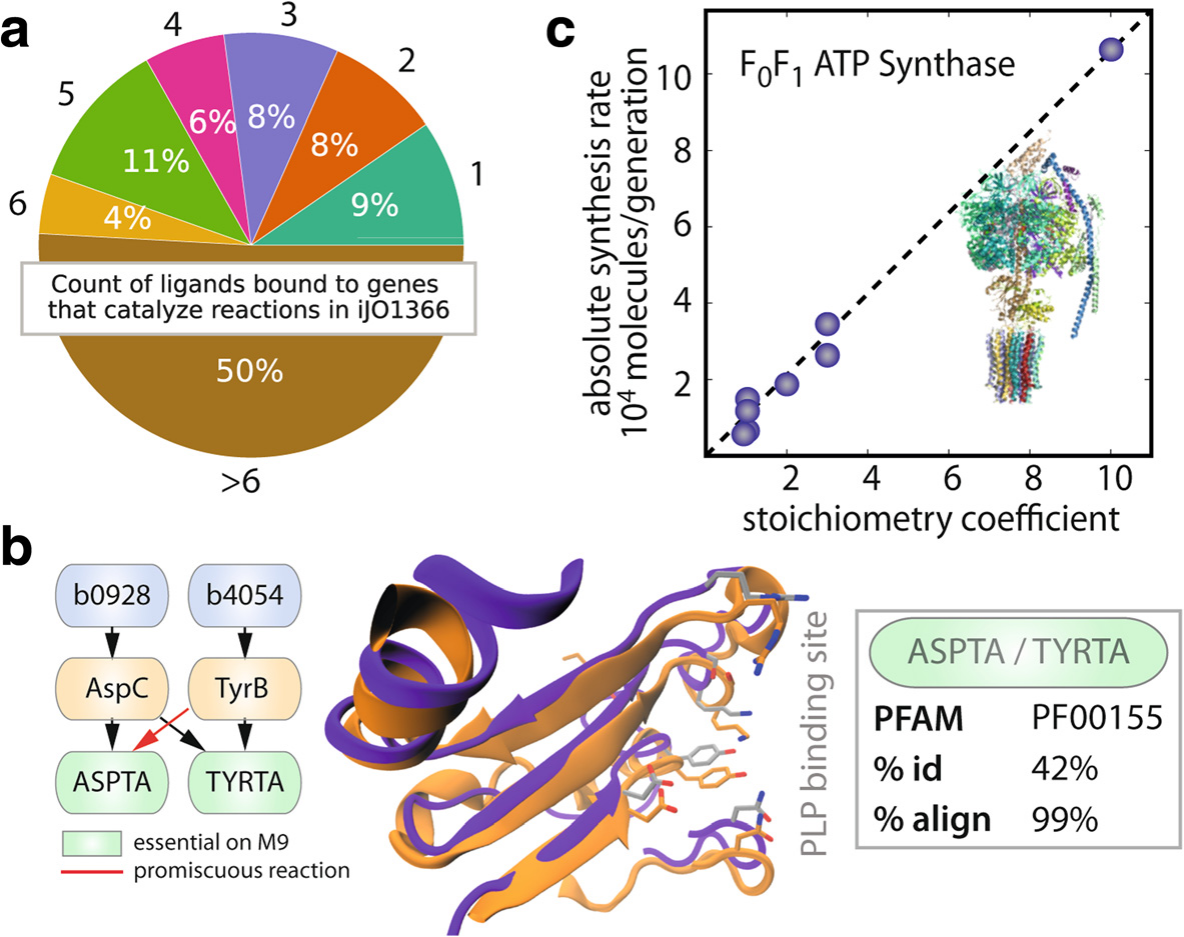
New structural systems biology applications using GEM-PRO. **a** The counts of different ligands from the Ligand Expo database (PDB) that are bound to holoenzyme protein structures in the *E. coli* GEM-PRO model and are linked to catalytic metabolic reactions. **b** An example of a highly promiscuous family of enzymes, transaminases, which have been shown to rescue the activity of another protein when its respective gene has been knocked out [6]. Pfam refers to shared protein fold family, '% id' refers to percent sequence identity, and '% align' refers to the 3D structural alignment of the two proteins. The plot in **c** demonstrates how the GEM-PRO model can be combined with experimental data, such as ribosomal profiling, to predict the in vivo abundance of proteins and their complex stoichiometry. The example shown here is that of ATP synthase, which indicates a high overlap between the complex stoichiometry stored in GEM-PRO and an experimental measurement

Many enzymes in the transaminase family are known to be capable of dual substrate recognition [39, 40]. Querying GEM-PRO, we find that aspartate aminotransferase, aspC (2.6.1.1), and tyrosine aminotransferase, tyrB (2.6.1.57), are both pyridoxal 5' phosphate (PLP)-dependent enzymes, share a common protein fold family (PF00155; Fig. 7b) and structurally align to give a high overlap of the substrate and cofactor binding sites. Structural properties such as these have been used to generate hypotheses about possible “underground” activities of enzymes, and some have been recently validated in vivo using an isozyme discovery workflow [6]. Extending the above analysis to the entire proteome, we are interested in addressing the question: “What is the degree of substrate specificity of proteins in a metabolic network?” Using the metabolic network models of *E. coli* and *T. maritima,* we find that both organisms have a subset of multi-functionality genes (i.e., genes that can catalyze more than one reaction); in *E. coli*, 4.4 % (60) of metabolic genes are involved in multiple enzymatic complexes and in *T. maritima*, over 19 % (90) are multi-functional. Although *T. maritima* has a higher degree of multifunctional peptides, the number of reactions with isozymes is consistent with that of *E. coli* (~30 %).

Protein structures of holoenzymes (i.e., proteins co-crystallized with cofactors or substrates/analogs) also provide a wealth of information on different protein-ligand interactions, as they can be directly compared to existing enzyme-substrate interactions in the metabolic network. We analyzed proteins bound to a representative set of compounds present in metabolism (e.g., not bound to glycerol, non-catalytic water molecules, or other types of detergents). To filter the large majority of these cases from the dataset, we classified the types of ligands bound to protein structures, which clusters ligands using a fast heuristic graph-matching algorithm [41, 42]. The type of ligand bound to a protein structure is grouped into different superclasses (e.g., lipids, amino acids, sugars, antibiotics), by comparing discriminating factors, such as the atom element, chirality, valence, and/or bond order (see Supplementary IPython notebook “Classify_PDB_Ligands.ipynb” and ref [41]). After filtering the ligands into metabolic (and non-metabolic) superclasses, we find 39 % of the total genes in the *E. coli* GEM-PRO model are representative holoenzymes (26 % of *T. maritima* genes). Surprisingly, we observe a large amount of metabolite binding versatility in *E. coli*, as 50 % of holoenzymes are experimentally shown to bind more than six different ligands (i.e., in different crystallographic structures of the same protein, see Fig. 7a). Each metabolite was described (according to its metabolite fingerprint similarity using Tanimoto coefficients [43]) and these coefficients were compared across the set of ligands bound to a given protein to determine the degree of variation in substrate specificity. We find that certain classes of enzymes, such as transferases (EC 2.-.-.-.), are only bound to very similar metabolites (which is consistent between *E. coli* and *T. maritima*), whereas lyases (EC 4.-.-.-.), are bound to the most structurally diverse set of substrates (see Additional file 1: Figures S13 and S14).

#### Protein complex stoichiometry predicts in vivo enzyme abundances

Does protein complex stoichiometry determine in vivo enzyme abundance? Previous work using ribosome profiling techniques revealed that multi-protein complexes have proportional synthesis rates [44]. This is both interesting and important because catalysis or activation of proteins is dependent on the proper complex formation of a specific number of homo- or hetero- subunits. Here, we apply a complementary approach, using genome-scale modeling of metabolism in conjunction with ribosome profiling data to identify which protein abundances are constrained by complex stoichiometry and which have higher free protein abundances.

Information about the stoichiometry (or ratio) of genes in the respective enzyme complex (and its functional properties) is found in organismal [45, 46] or protein databases [47] and can be directly incorporated into GEMs (e.g., in the annotated gene-protein-reactions, or GPRs). GPRs link a set of genes to the metabolic enzyme to the catalyzed reaction, providing a starting point for the reconstruction of enzyme complex stoichiometry. In this section, we discuss how to predict enzyme abundances, identify peptides that are not expressed stoichiometrically, and predict the partitioning of peptides across the multiple complexes to which it belongs. To associate the metabolic reactions with structures of their catalyzing enzymes, we integrated GEM-PRO together with the genome-scale models of metabolism and expression (ME-model) for *E. coli* [48]. The coverage of complex stoichiometry is relatively complete (95 %). We find that the majority of metabolic enzymes are homomers (90.3 %), for which, we see a strong preference for even stoichiometry. This is consistent with general trends among homomeric complexes towards even stoichiometry, and has been explained based on the ability of complexes with even stoichiometry to form complexes with dihedral symmetry as well as rotational symmetry [49]. Furthermore, we find that 4.4 % (60) of metabolic genes are involved in multiple enzymatic complexes and 30 % of reactions are catalyzed by isozymes.

Coupling information from genome-scale reconstructions, known enzyme complex stoichiometry, and ribosomal profiling data, we can predict in vivo protein abundance in *E. coli.* As depicted in Fig. 7c, this novel framework can be applied to identify and predict protein complex stoichiometry [44, 50]. As illustrated in the Supplementary IPython notebook, "Complex_Stoichiometry.ipynb", protein complex stoichiometry can be converted into a computable (mathematical) format for validation with experimental ribosomal profiling data [44, 51]. A protein stoichiometric matrix is assembled in which the rows represent proteins, the columns represent enzymes, and the entries indicate the stoichiometry of the protein within the enzyme (akin to a stoichiometric matrix of metabolism, used in GEMs [5]). This matrix, combined with quantitative data on protein expression [52, 53], can then be used to determine feasible enzyme (and free peptide) abundances using constraint-based modeling methods [54] and available software [55, 56]. We find that the maximal and minimal enzyme abundances, computed using flux variability analysis (assuming free peptide abundance is minimized) indicate that enzyme abundances are quite constrained by stoichiometry alone (see Additional file 1: Figure S15). Interestingly, we find that many of the proteins with the largest free abundances are periplasmic substrate binding proteins (see Additional file 1: "Complex_Stoichiometry.ipynb"). These proteins are not always in complex with the transporter protein itself and, therefore, are not produced stoichiometrically with the rest of the transporter complex, making their abundances less constrained.

#### Comparative systems biology of different bacterial proteomes

To date, there has been a great deal of attention placed on understanding the genetic differences between *T. maritima* and other Eubacteria [57–63]. Whole-genome similarity comparisons indicate that *T. maritima* is the most Archaea-like organism compared to other eubacterial species [57–63], with 24 % of genes appearing to be more closely related to archaeal genes [63, 64]. Less attention, however, has been focused on characterizing the differences between proteomes of species. Of the studies that evaluate protein-level differences, many have focused on families of proteins [65, 66], and few have focused on comparing proteins that span across entire metabolic networks. The novelty of using GEM-PRO for comparative studies is the ability to map genes to their gene products (proteins) to the reactions they catalyze within a single database. Such a mapping allows for high-level structural comparisons of *functionally* relevant sets of genes: homologous genes, genes that catalyze more than one reaction (i.e., promiscuous), genes that catalyze similar reactions (i.e., isozymes) and genes with high sequence or structural similarity. Here, we apply GEM-PRO to address the question, “How different are bacterial proteomes and what are the main properties that distinguish them?”

The first notable difference, when comparing GEM-PROs of *E. coli* and *T. maritima,* is the spread of molecular motifs across metabolic proteins, which greatly distinguishes the two proteomes from one another. We used the Flexible structure AlignmenT by Chaining AFPs (Aligned Fragment Pairs) with Twists (FATCAT) [67] algorithm to detect all of the aligned fragment pairs (AFPs), based on previous PDB-wide alignment of representative protein domains [68]. The observed AFPs are regions of a protein that cluster based on similarities in local geometry and take into consideration protein flexibility by clustering regions of the protein that can undergo different geometric transformations. Considering all proteins in both the *E. coli* and *T. maritima* GEM-PRO models, we found a total of 874 and 197 unique domains (according to SCOP or PDB-based annotations), respectively, which span the whole of metabolism (i.e., 1819 total protein structures). We find that 36 domains are shared between *T. maritima* and *E. coli* (see Additional file 1: for more details). Furthermore, comparing the distribution of complex stoichiometry between *E. coli* and *T. maritima*, we find that for both organisms, the majority of metabolic enzymes are homomers (90.3 % and 71.1 %, respectively).

**Fig. 8:**
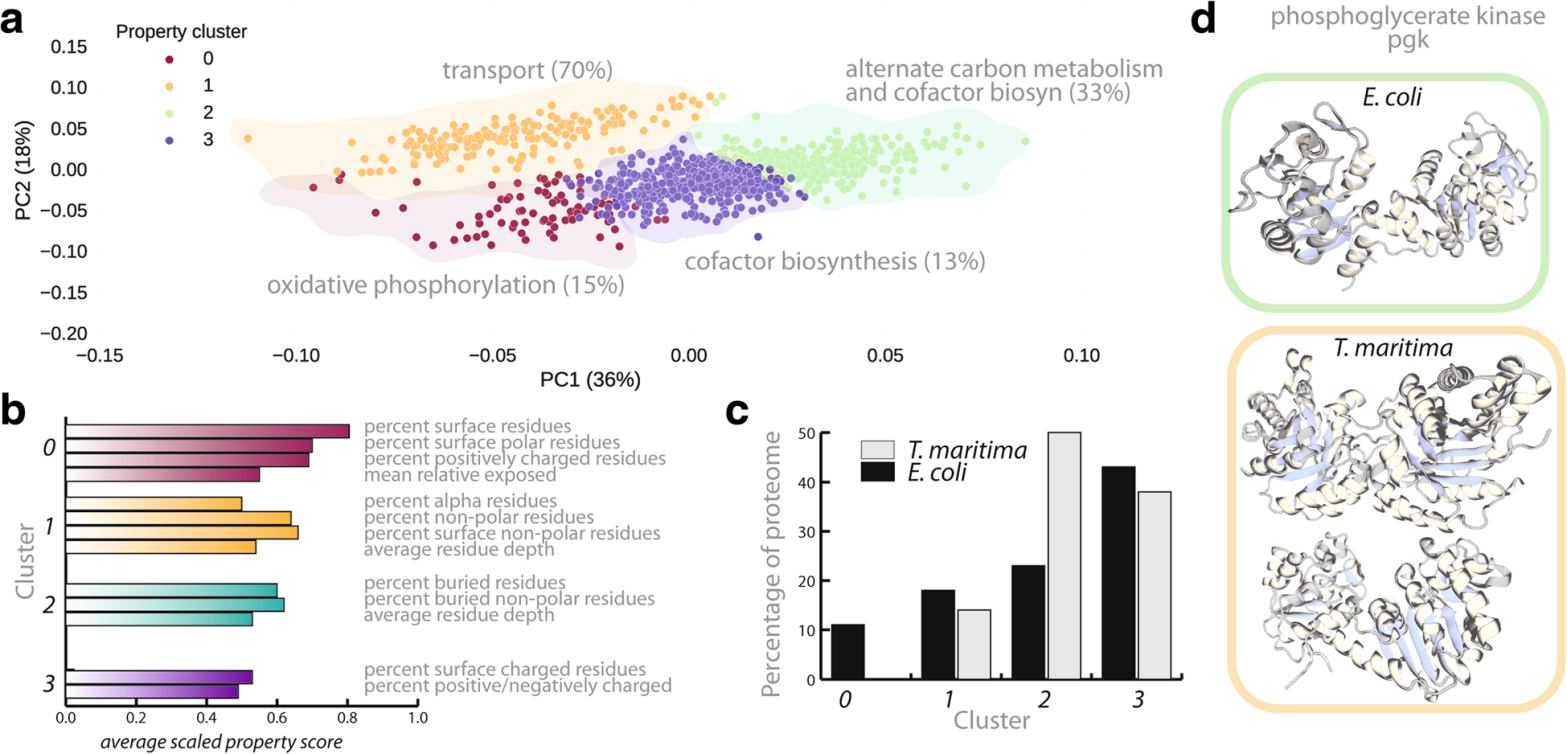
In **a**, K-means clustering of all *E. coli* and *T. maritima* protein structural properties (29 features, including SASA, percent polar, nonpolar, buried, surface, charged residues and others). The K-means clustering algorithm clusters all proteins into four distinct clusters (based on the percent variance explained per cluster using the elbow method, see Additional file 1). Interestingly, metabolic subsystems in *E. coli* show distinct structural characteristics in their respective proteins. The subsystem with the most proteins in a given cluster is reported. In **b**, we report the main structural characteristics that distinguish proteins across clusters. The numbers represent averaged scaled property values across all proteins within a given cluster (see Additional file 1). The property values generally represent the percentage of the protein that is described by a given property (e.g., percentage of the protein which is nonpolar). In **c**, the percentage of *E. coli* and *T. maritima* proteomes within each cluster are shown. Surprisingly, certain clusters are enriched in *E. coli* proteins (cluster 0) and certain in *T. maritima* proteins (cluster 2). Total numbers of proteins in each cluster are 154, 318, 592, and 763 for cluster 0–4, respectively. In **d**, an example of a homolog (pgk) which is present in entirely different clusters (cluster 2 for *E. coli* and cluster 1 for *T. maritima*). The structural differences can mainly be explained by the fact that in *T. maritima*, pgk (PDB 1VPE) is fused with tpi (PDB 1B9B), creating a protein which is triple in length to that of its *E. coli* counterpart (PDB entry 1ZMR)

To understand whether the properties of entire proteomes are distinguishable between organisms, we carried out PCA on 29 computed secondary structural properties (see Additional file 1: Table S5 and Fig. 8a). The projections of the first two principal components explain 60 % of the normalized property distribution. Using K-means clustering, we find that protein properties separate into four discrete clusters (based on the percent variance within clusters as detailed in Additional file 1). The main difference between the clusters of proteins is the percent composition of secondary structural elements, such as *α*-helical and *β*-extended strand, solvent-accessible surface area and percentage of charged residues (Fig. 8b). For example, in one cluster (‘1’), 64.7 % of amino acids are found in *α*-helices. A correlation matrix derived from the properties of proteins in this cluster indicates that the majority of residues found in *α*-helices also have higher percentages of hydrophobic content while other residues found in *β* strands are highly charged. The majority (155 out of 247) of this cluster of proteins are membrane-bound proteins, which are known to have distinguishing exterior domains [69, 70], and correlate based on a preference for *α*-helices and a neutral surface charge, compared to those proteins in other clusters.

As illustrated in Fig. 8c, the percentage of the proteome in each of the four clusters differs between organisms; certain clusters are present (or enriched) in only one of the organisms (such as cluster 0 for *E. coli* and cluster 2 for *T. maritima*). Comparing the unique aspects of proteins within each of the clusters, we find that certain characteristic features distinguish proteins based on their metabolic roles as well as based on which organism they belong to. For most clusters, proteins belong to a single (or a select few) subsystem(s), which suggests that these features may play a role in self assembly and cellular localization. For example, comparing the second and third clusters (1 and 2), many of the members (over 70 %) function as transport proteins versus alternative carbon metabolism and cofactor biosynthesis (33 %). For differences between proteomes, we find that the first cluster (0) consists of only *E. coli* proteins (Fig. 8c), which are enriched in surface-exposed residues and tend to be polar or positively charged (Fig. 8b). However, in the third cluster (2), we find an increased number of thermophilic proteins compared to the number of mesophilic proteins with a higher degree of buried, nonpolar residues, and are less polar and solvent accessible. This is consistent with what is generally known about protein stability [71], such as those dominated by forces that drive protein folding (e.g., the burial of nonpolar groups, increased number of hydrophobic interactions and decreased solvent accessibility).

#### Characterization of proteins with growth rate-limiting reactions at high temperatures

High temperatures impose a heavy burden on organisms with respect to the functioning of cellular metabolism. Understanding the molecular basis for stability is necessary to grasp the the fundamental nature of protein structure as well as to engineer high-temperature industrial processes [72]. In general, structure-based analyses have been used to discover properties of thermostability [71, 73–75], however, there remains a significant challenge to pinpoint which characteristic features of proteins lead to detectable differences between thermophiles and mesophiles [76, 77]. Using an entirely different approach, genome-scale models of metabolism point to specific proteins that limit the ability of the cell to grow and function at a given temperature [17]. For example, specific *E. coli* proteins, identified as “hotspots,” are linked to reactions in the metabolic network that limit or diminish the cellular growth rate at higher temperatures (e.g., due to protein unfolding/degradation). The novelty of this approach is that we can hypothesize which “hotspot” proteins are under selective pressure (on the basis of how important their function is to the entire metabolic network) and require adaptation to function at higher temperatures. 

Here, we are interested in the characterization of molecular properties of *T. maritima* homologs that set them apart from their *E. coli* counterparts, potentially allowing for functional proteins at higher temperatures. To begin, we focused on hotspot proteins in *E coli*, which are known to be growth-rate limiting at high temperatures. To identify  *T. maritima* homologs within the subset of hotspot proteins , we took advantage of the extensive database of both GEMs to effectively map between *E. coli* and *T. maritima* genes that have a similar sequence and metabolic function (a total of 219 homologs; see Additional file 2: "Database S2: Table 01"). In this case, we clustered alignments of *E. coli* with *T. maritima* PDB templates into three classes (high, medium, and low-medium overlap) based on the root-mean-squared-deviation (RMSD) of the protein backbones (less than 5 Å, 5–7 and 7–10 Å, respectively) and an alignment coverage of greater than 70 % of the total length of the protein. Surprisingly, we find that, out of 219 homologs, only 10 % (19) of *E. coli* genes share a structurally similar domain with their *T. maritima* homologs (all cases align with RMSD < 5 Å). Of the 10 % that are structurally similar, we linked their respective metabolic functions to amino acid biosynthesis, cofactor biosynthesis, or cell envelope biosynthesis. A few cases related to tRNA and methionine metabolism also show a high degree of structural similarity, despite low nucleotide sequence identity (e.g., b3559/TM_0216 have 30.1 % sequence identity and b4019/TM_0269 have 27.8 % sequence identity).

Particularly interesting cases pulled out from this analysis are those of 3-phosphoglycerate kinase (pgk, EC 5.3.1.1) and the *b* subunit of atp synthase (atpB, EC 3.6.3.14). Comparing the extremely stable thermophilic pgk with its less stable, mesophilic homolog reveals that this peptide correlates to proteins in cluster 2, whereas the thermophilic pgk correlates to proteins in cluster 1. The crystallographic structure of the thermophilic pgk shows increased rigidity from the many intramolecular contacts, alpha helices, and loop regions [78] consistent with cluster 1 properties. Furthermore, the size of the *T. maritima* pgk is three times that of its *E. coli* counterpart (280 kDa versus 43 kDa), as it is a tetrameric fusion protein (pgkfus) of two enzymes, namely pgk and triosephosphate isomerase (tpi, 2.7.2.23), illustrated in Fig. 8d, bottom. Despite a difference in relative enzyme efficiency, the fusion protein is active when previously cloned and expressed in *E. coli*, confirming the authenticity of the two separable proteins and enzyme activities resulting from this gene in the mesophilic host [79]. In this context, covalent fusion of two proteins to complexes or assemblies might represent an additional stabilization strategy, particularly for “hotspot” enzymes that become unstable at higher temperatures, like pgk.

A structural comparison of the β subunit of ATP synthase polypeptides indicates that the *T. maritima* protein has a higher degree of buried, nonpolar residues that, on average, are less solvent exposed (i.e., a larger average residue depth of the alpha carbon atoms in the protein). In contrast, the *E. coli* peptide is much more solvent exposed and its residues are, on average, more polar or positively charged. A previous study, which characterized the chimeric soluble β polypeptides in vitro showed that the *T. maritima* protein melted cooperatively with a midpoint more than 20 °C higher than that of the *E. coli* sequence [80]. The study revealed the effects of substituting different sequences in the *E. coli* peptide, showing which parts of the peptide tolerated the most change without a loss of function and which changes led to an increased thermostability. The structural differences brought out by this pairwise comparison are consistent with the fact that the average relative contact order (which correlates to solvent accessibility) of *T. maritima* proteins is significantly different than their close mesophilic homologs [77].

### Dissemination of GEM-PRO and development of new training resources

Equally important to providing higher quality models is providing the community with complete knowledge bases, tools, and training examples for the continuous development of genome-scale modeling approaches. Historically, advances in genome-scale modeling have been accelerated by the wide dissemination of network reconstructions, modeling methods, and their continual curation and updating to incorporate new information. Furthermore, as GEM-PRO enables modeling of cellular processes that span a wide range of biological, chemical, and structural detail, input from different scientific disciplines could vastly enhance the capabilities of current methods and approaches used in systems biology. To make GEM-PRO accessible to a wide-range of scientific backgrounds, we present GEM-PRO workflows for these two contemporary organisms, *E. coli* and *T. maritima*.

As Additional file 1, we describe how various protein-related data types are paired with GEMs (Fig. 6). We provide bioinformatics scripts together with tutorials (in the form of IPython notebooks) as Additional file 1 to explicitly describe how protein-related information can be linked to genome-scale models to study: (i) the evolution of protein fold families in metabolism; (ii) temperature-dependent growth rate predictions; (iii) the diversity in protein-ligand interactions in a metabolic network; (iv) the organization of protein complex stoichiometry and how it can be paired with ribosomal profiling data to describe in vivo protein abundance.

## Conclusion

Protein structures and their molecular assemblies offer a wide range of possibilities to further enhance the predictive scope of genome-scale modeling by providing information on the sequence of molecular events in a pathway, how to interfere with a pathway to treat a pathology, or the evolutionary history of contemporary organisms. The further integration of protein-related data into metabolic network reconstructions will rely on clear mapping protocols and the development of bioinformatics tools that will aid in this process. This contribution, the bioinformatics tools, and the accompanying tutorials, which are based on constraint-based modeling methods through COBRApy [56], describe the generation and application of GEM-PRO models. Here, we have shown the utility of integrating molecular scale analyses with systems biology approaches by discussing several comparative analyses on the temperature dependence of growth, the distribution of protein fold families, substrate specificity, and characteristic features of whole cell proteomes.

The dissemination of the GEM-PRO modeling framework is likely to broadly impact work in a wide array of disciplines, including structural biology, computational chemistry, systems biology, and biotechnology. The ability to characterize the structural, chemical, and binding characteristics of metabolic proteins in different organisms also enables the further development of *in silico* tools capable of identifying isozyme activity on a genome-scale. Recently, a number of studies have emerged [6, 81, 82] that have used genome-scale models together with complementary bioinformatics techniques to characterize the versatility of enzymes on a systems level. Such studies can easily be extended to include the assessment of protein structural data and can be used to complement current “gap-filling” methods [83, 84] for model improvement. Current “gap-filling” methods typically use amino acid sequence identity as a measure for predicting enzyme similarity. However, some candidates are likely to be overlooked, since proteins with low sequence identities (e.g., <15 % in the globin family) have also been shown to share similar folds and functions [65, 85, 86]. Evaluating the capacity of a protein to catalyze more than one reaction is also especially important to applications in metabolic engineering [87–89], where such proteins serve as an ideal starting platform for engineering novel capabilities as well as increasing substrate specificity.

Finally, GEM-PRO models offer insight into the physical embodiment of an organism’s genotype and provides a new way to compare genomes by linking genes to their encoded gene product, to the protein’s structure, and finally, to the reaction catalyzed by that protein (or its molecular assembly). The use of GEM-PRO models as a comparative systems biology approach demonstrates that important aspects of the functional differences between organisms (e.g., due to lifestyle changes) are not only derived from differences in their genetic components but also from the physical interactions of their molecular components. Together with previous applications on the phylogenomic analysis of protein structure [90], global motifs on protein fold and domain architecture [91, 92], and evolution of modern metabolism [7, 93], mapping the properties of proteins to their respective genes offers a novel perspective of the molecular, biochemical, and phenotypic features of contemporary organisms. This comparative framework enables exploration of adaptive strategies for these organisms and opens the door to many new lines of research, including metabolic engineering and the design of thermostable enzymes.

## Methods

### Data retrieval and manipulation

Incorporating protein-related information into a GEM involves four stages of semi-automated curation: (i) map the genes of the organism to available experimental protein structures, found in publicly available databases, such as the Protein Data Bank (PDB); (ii) determine genes with and without available protein structures and perform homology modeling using the I-TASSER suite of programs [30] to fill in gaps where crystallographic or NMR structures are not available; (iii) perform ranking and filtering of PDB structures for each gene based on a set selection criteria (e.g., resolution, number of mutations, completeness); (iv) map GEM genes to other databases (e.g., BRENDA [94, 95], SwissProt [96], Pfam [97], SCOP [98]) for complementary protein-structure derived data. The quality of the reconstruction expansion process to include high confidence protein structures is considered by carrying out a series of QC/QA verification steps during the ranking and filtering stage. The GEM annotation of the organism of interest is stored in SBML and Matlab formats and many organisms can be found in the BiGG database [5]. Amino acid sequence of the proteins of interest are stored in FASTA format. To map protein structural data to a GEM, we make use of Python modules, ProDy [99, 100] and Biopython [101] to parse information in the PDB files. The molecular visualization software VMD [102] was used for viewing the 3D structure of the modeled protein and the predicted functional sites and the creation of images. Installation of PfamScan and HMMER3 algorithms are required for generating protein fold families for certain proteins [103, 104]. Open source software for protein structural predictions are available and are used in conjunction with the IPython framework.

### Data organization into IPython Notebooks

In the Supporting Information, we provide discrete examples of how to use the expanded metabolic network reconstructions with protein information to predict cellular phenotypes, which include (i) the discovery of multimeric properties of metabolic enzymes; (ii) the predicted growth of *E. coli* at different temperatures; (iii) predicting the effects of antibacterial drugs in *E. coli*; (iv) the discovery of patterns in fold families distributed across the metabolic network in *E. coli* and (v) the discovery of ligand similarity and potential for promiscuity in the metabolism *E. coli*. The tutorials provided in Supporting Information are designed in such a way that aids the user to properly access information in the GEM-PRO database, easily reproduce previously reported findings and organize information into meaningful representations. The main objective of the designed framework is to assist in (i) mapping between useful and unique identifiers; (ii) locate and query various data sources and (iii) identify fruitful and meaningful associations between the disparate datasets. We provide tutorial-like IPython notebooks as a means to organize the output of the database into easily manageable and understandable modules. Such a framework is the first of its kind for constraint-based modeling and provides full details that can be reproduced and updated as new data becomes available. For more details, see the Additional file 1.

### Homology modeling framework

The I-TASSER protocol is described by the following steps: (i) for each protein of interest, homologous templates are identified and used to assemble the queried protein; (ii) modified Monte Carlo based replica exchange simulations are performed to cluster the lowest-free energy states of the assembled structure; (iii) the fragment-based assembly simulation is performed a second time to further refine the model and remove steric clashes; (iv) the function of the query protein is inferred by structurally matching the predicted 3D models against the proteins of known structure and function in the PDB. In order to assess the quality of the predicted structure, the accuracy is predicted from a confidence score (C-score or TM-score), which is defined based on the quality of the threading alignments and the convergence of the assembly refinement simulation used in steps ii and iii. I-TASSER is capable of generating multiple model predictions with a rank-ordered C-score. For more details about I-TASSER, please refer to the published literature [28].

### Prediction of Pfam family folds (HMMER)

The database currently maintains 14,831 manually curated entries in the current release and is accessible via web servers (http://pfam.sanger.ac.uk/ and http://pfam.xfam.org/). This information allows for the classification of proteins via amino acid sequence into distinct protein families who share domain architecture through the HMMER suite of programs [105]. The challenges of predicting protein families using HMMER3 are discussed elsewhere [106]. For the genes in our models without Pfam annotations, we have run the freely available HMMER source code [103, 104] to fill in the “gaps” in the Pfam knowledgebase.

### Temperature-based Predictions in the *E. coli* Metabolic Network

Temperature-related properties of proteins (e.g., melting point temperature or T_M_) were determined using both experimental and predicted values for the melting temperatures of proteins. The two main sources of this experimental data were taken from ProTherm [107] and BRENDA [95] online data services. By querying ProTherm and BRENDA temperatures of specific metabolic proteins were linked to metabolic genes via their respective EC number. In the Additional file 1, we have provided a script that performs the direct mapping between Blattner number and EC for querying both ProTherm and BRENDA databases (see the Supplementary IPython notebook titled, "Predicting Growth Rate at Various Temperatures"). For the *i*JO1366 model of *E. coli*, we find low coverage of temperature related data (only 29 out of 1366 genes with automated querying and 193 genes with semi-automated and manual curation). Thus, the experimentally determined T_M_ values were supplemented with predicted T_M_ using a previously published method [108]. We provide an example of one out of the four bioinformatics-based computational prediction of T_M_ which derived from the amino acid sequence.

### Reconstruction of Protein Complex Stoichiometry

We updated the reconstruction of complex stoichiometry of enzyme complexes that catalyze metabolic reactions to include over 500 new complexes. We have included the list of added reactions together with the nearly complete mapping to complex stoichiometry in the Additional file 1. Metabolic models contain gene-protein-reaction relationships (GPRs), which are boolean statements on the requirements of genes for catalysis. However, more detailed reconstructions that include protein structures and models of metabolism and protein expression (ME-Models) benefit [48, 109] from information on enzyme stoichiometry. While the previous versions of GEM-PRO [7, 110] included information on single protein chains and protein complexes (using information both experimentally determined and putative PISA predictions [111]), the updated GEM-PRO extends the coverage to include additional data derived from experimentally determined enzyme complex stoichiometry. There are several additional sources of data on the stoichiometry of proteins in complexes, including PDB structures and protein gels; much of this data is already compiled in databases such as Ecocyc [45, 46] or UniProt [47]. Experimentally determined structures and structures from homology modeling were used to achieve 93 % structural coverage of proteins in the *i*JO1366 network and between 24 % and 33 % coverage of protein-substrate binding conformations. Manual curation for enzymes and metabolic reactions that do not perfectly match between the M-Model and databases is necessary. This procedure was performed by O'Brien et al. [48] starting from the *i*JO1366 metabolic model and mapping to the enzyme annotation in EcoCyc [45].

### Calculation of Protein 3D Structural Properties

We calculate 29 physical properties of the protein to construct a multidimensional data matrix, including solvent-accessible surface area (SASA), number of total contacts, disulfide bond distance (SS-bond), percent of the protein that is buried, percent of the protein that is on the surface, secondary structure composition (*α*−helical content, *β*−strand content, 3^10^ helix content, *π*−helix content, hydrogen bonded turn content, bend content, disordered content), ovality (SASA/N_res_^2/3^), residue depth (distance of the C atom from the protein surface), percent of the total structure that is nonpolar, polar, positively charged, or negatively charged, and percentage of the surface/buried residues that are nonpolar, polar, positively charged, or negatively charged. SASA was calculated according to the algorithm of Lee and Richards [112, 113] with a probe radius of 1.4 Å. Residues with a SASA measurement greater than 3 Å^2^ are assigned as surface residues. The residue depth has been calculated for all atoms in the entire protein based on Michel Sanner's Molecular Surface (MSMS) method [114] and is evaluated from the average distance of all atoms to the surface of the protein. The number of disulfide-bonds is calculated from the 3D coordinates of sulfur atoms (using a 5 Å bonding distance cutoff).

## Availability of data and materials

Database S1: *E. coli*. Excel file containing GEM-PRO related information for *E. coli* (Additional file 3).

- Table 01: GEM-PRO master dataframe. All reactions, genes, sequence and structure ID mappings.
- Table 02: Enzyme complex information for the associated reaction.
- Table 02a: Updates to the previous complex information available in 2013.
- Table 03: 3D structural properties of all representative structures per gene.
- Table 03a: 3D structural properties of all homology models.
- Table 04: PFAM retrieved and computed properties.
- Table 05: Structural quality of PDB structures, including PSQS and PROCHECK scores.
- Table 06: Structural quality of homology, including TM-scores, C-scores, PSQS, and PROCHECK scores.

Database S2: *T. maritima*. Excel file containing GEM-PRO related information for *T. maritima* (Additional file 2).

- Table 01: GEM-PRO master dataframe. All reactions, genes, sequence and structure ID mappings.
- Table 02: Enzyme complex information for the associated reaction.
- Table 03: 3D structural properties of all representative structures per gene.
- Table 04: PFAM retrieved and computed properties.
- Table 05: Structural quality of PDB structures, including PSQS and PROCHECK scores.
- Table 06: Structural quality of homology, including TM-scores, C-scores, PSQS, and PROCHECK scores.

Dataframes, mapping files, analysis scripts, tutorials and other documentation have been uploaded to a public Github repository and are available at: https://github.com/SBRG/GEMPro/tree/master/GEMPro_recon/.

Four iPython tutorial notebooks are hosted in the same Git repository and are available for viewing:

I. Understanding evolutionary relationships of fold families in metabolism: https://github.com/SBRG/GEMPro/blob/master/GEMPro_recon/Ecoli/tutorials/Protein_Fold_Familes.ipynb, https://github.com/SBRG/GEMPro/blob/master/GEMPro_recon/Tmaritima/tutorials/Protein_Fold_Familes.ipynb.
II. Predicting growth rate at various temperatures: https://github.com/SBRG/GEMPro/blob/master/GEMPro_recon/Ecoli/tutorials/Temperature_Dependent_Growth_Prediction.ipynb.
III. Classify and characterize the co-crystallized ligands in GEM-PRO: https://github.com/SBRG/GEMPro/blob/master/GEMPro_recon/Ecoli/tutorials/Classify_PDB_Ligands.ipynb, https://github.com/SBRG/GEMPro/blob/master/GEMPro_recon/Tmaritima/tutorials/Classify_PDB_Mols.ipynb.
IV. Protein complex stoichiometry for M-Model enzymes: https://github.com/SBRG/GEMPro/blob/master/GEMPro_recon/Ecoli/tutorials/Complex_Stoichiometry.ipynb, https://github.com/SBRG/GEMPro/blob/master/GEMPro_recon/Tmaritima/tutorials/Complex_Stoichiometry.ipynb.

## Additional file

Additional file 1: Supplementary information. (PDF 2299 kb) Additional File 2: Database S2:
*T. maritima.* Excel file containing GEM-PRO related information for *T. maritima*. (XLSX 775 kb) Additional File 3: Database S1:
*E. coli.* Excel file containing GEM-PRO related information for *E. coli*. (XLSX 13.3 mb)
