# Supplementary material for: Systems biology of the structural proteome
Source: BMC Syst Biol. 2016 Mar 11;10:26. doi: 10.1186/s12918-016-0271-6 (PMC4787049; doi:10.1186/s12918-016-0271-6)
Supplement: Additional file 1: — Supplementary information. (PDF 2299 kb) [file 12918_2016_271_MOESM1_ESM.pdf]

Systems Biology of the Structural Proteome

Supplementary Information

Elizabeth Brunk[a,b, †], Nathan Mih[c, †], Jonathan Monk[a], Zhen Zhang[a], Edward J. O'Brien[a], Spencer E. Bliven[c,d], Ke Chen[a], Roger L. Chang[e], Philip E. Bourne[f], Bernhard O. Palsson*[a]

* Correspondence should be addressed to: B.O.P. (palsson@ucsd.edu)
[a] Department of Bioengineering, University of California, San Diego, La Jolla, CA 92093
[b] Joint BioEnergy Institute, Emeryville CA, 94608
[c] Bioinformatics and Systems Biology Program, University of California, San Diego, La Jolla, CA 92093
[d] National Center for Biotechnology Information, National Library of Medicine, National Institutes of Health, Bethesda, MD 20894
[e] Department of Systems Biology, Harvard Medical School, Boston, MA 02115
[f] Office of the Director, National Institutes of Health, Bethesda, MD 20894
† Authors contributed equally

Contact information
Elizabeth Brunk: ebrunk@ucsd.edu
Nathan Mih: nmih@ucsd.edu
Jonathan Monk: jmonk@ucsd.edu
Zhen Zhang albertzhang017@gmail.com
Edward J. O'Brien: ejobrien@ucsd.edu
Spencer E. Bliven: sbliven@ucsd.edu
Ke Chen: kec003@eng.ucsd.edu
Roger L. Chang: roger_chang@hms.harvard.edu
Philip E. Bourne: philip.bourne@nih.gov
Bernhard O. Palsson: palsson@ucsd.edu

Table of Contents

- Generation and Updating GEM-PROs through a Systematic Pipeline
  - Mapping the PDB to a GEM
    - E. coli
    - T. maritima
  - Homology Modeling
  - QC/QA Procedure
  - Model Refinement
- Model Comparisons
  - Mapping the PDB to a GEM
    - E. coli
    - T. maritima
  - Homology Modeling
    - E. coli
    - T. maritima
  - QC/QA Procedure
    - E. coli
    - T. maritima
  - Model Refinement
    - E. coli
    - T. maritima
- Dissemination of GEM-PRO and Development of New Training Resources
  - Protein Fold Families in Metabolism
    - E. coli
  - Growth at Different Temperatures
    - E. coli
  - Metabolite Versatility in co-crystallized complexes
    - E. coli
  - Enzyme Abundances and Protein Complex Stoichiometry
    - E. coli
- Comparative Systems Biology of Different Species
- References

Generation and Updating GEM-PROs through a Systematic Pipeline

The need for continuous updating of GEM-PRO models is evident based on the fact that the number of 3D biomacromolecules deposited in publicly available databases continues to increase exponentially each year [1]. The updated GEM-PRO modeling framework provides an automated pipeline for querying online servers and databases that contain protein-related information to construct high-quality, reproducible and reliable GEM-PRO models, starting from any metabolic model. By constructing such a pipeline, we had to overcome a number of challenges, such as the selection of reliable, organism-specific identifiers to enable efficient mapping and the development of a quality control screening technique to determine which structures were high enough in quality to be used in the model. In this section, we discuss the results related to the mapping of protein structures to genes and provide details related to the quality of these structures in the section “Quality control and quality assessment of all structures.

One of the main goals of this contribution is the creation of new models that can be easily queried, mapped onto a metabolic network, and linked to existing constraint-based modeling techniques [2] (e.g., COBRApy [3]. Following the workflow displayed in Figure 2, we have organized the discussion of all updates to the GEM-PRO modeling framework based on the five stages of the pipeline: (i) assessing metabolic gene coverage of structures; (ii) the integration of standard, high-quality I-TASSER homology models [4]; (iii) the quality assessment of all structures and homology models; and (iv) model refinement of structural and sequence-based properties. Each of the above points is discussed in further detail in the following sections.

Mapping the PDB to a GEM

In the first step of this “structural reconstruction”, the main goal is to automate the querying of online servers and databases that contain protein structural information and other protein-related information and effectively link this data to the metabolic network reconstruction. The main question that is addressed in this section is, “What is the most effective way to link information stored in a GEM to information stored in different protein-related databases?” Several challenges arise at this stage, which will be further elaborated on below, which include selecting an appropriate identifier for mapping, dealing with redundancy in the data frame output, and the reliability of the information being mapped to the metabolic network reconstruction.

The input of our semi-automated mapping procedure is a high-quality metabolic network reconstruction that should be in a standard, compatible format, such as the Systems Biology Markup Language (SBML) or a Matlab file [5]. Previously published metabolic network reconstructions are readily available [6]. The main outputs of the mapping include correct UniProt accession codes and PDB entries, sequence information, and structural information [7,8].

At this stage, all information is passed into the Pandas Python module [9,10], which has proven to be a useful open source tool capable of efficiently organizing large-scale data into a so-called “data frame”, similar to a queryable SQL table. Throughout this protocol, we will be referring to the organization of the metabolic network reconstruction into the data matrix as the “master data frame”. The master data frame initially contains information from the original metabolic network reconstruction, including the gene IDs, the metabolic reaction catalyzed, the metabolites involved in the reaction, etc. This information is obtained utilizing the COBRApy Python module [3].

Another challenge that arises in this stage is understanding how to query various databases and get the maximum amount of available data for a set of genes. The manner of mapping and the use of specific identifiers are different for each database and must be carefully selected before commencing the mapping process. For example, different identifiers (e.g. gene identifier, gene name, EC number, UniProt accession number, etc.) might be required for the querying of particular databases (KEGG, Entrez, BioCyc databases, or manual annotation from genomics datasets) in order to gain maximum coverage or non-ambiguous mapping of genes in a GEM model. Moreover, the list of candidate genes to map data to may not necessarily be complete or comprehensive, as many ambiguous identifiers might be present in the list. For example, proteins may not have well defined EC numbers, but their functions are considered in metabolic reconstructions. (e.g. a gene has not been assigned an identifier) or entirely unambiguous (e.g. a gene is assigned a non-specific identifier, such as EC i.j.k.-). Thus in this step it is necessary to ensure that a suitable identifier is used.

.It is important to note that a gene and its respective gene product may have a number of biological roles and may co-complex with numerous gene-products to catalyze more than one reaction. For example, a reaction may be comprised of a single functional chain (homomer) or multiple chains (gene products) to form a functional heteromer or multimeric complex. Genes may encode multiple isozymes, which serve a given purpose under a particular environmental condition. As a result, each reaction can have multiple representative structures, which leads to redundancy in the master data frame. We approach this challenge in two different ways: (i) we make use of Gene-Protein-Reaction rules (GPRs), which are a part of the original reconstruction and (ii) we provide examples of how to query the master data frame in the Supporting Information. For the first case, GPRs provide information detailing the number of different gene(s) required to catalyze a reaction or, whether multiple genes catalyze the same reaction independently. For both cases, multiple row entries in the metabolic network reconstruction may appear to be linked to the same PDB entry. For the second case, we have provided discrete examples in the Supplementary IPython notebooks to provide a tutorial-like examples of how to properly query the data frame to avoid redundant information

The final challenge at this stage is to evaluate how confident we can be in the information that has been mapped to the metabolic network reconstruction. There is a spectrum of quality to consider, which includes if the entries have been manually curated or reviewed (for higher quality), whether the names or identifiers of the PDBs have changed or become obsolete, or whether the structures mapped through UniProt are theoretical or experimental. For example, for certain genes with a low PDB coverage, it is advantageous to include UniProt entries that have not been reviewed or may contain theoretical structures. Therefore, during this mapping process, we have also kept track of where the data is coming

from (i.e. which database source) and how confident we are in its quality. This information we later use for validation, cross-checking and reproducibility purposes. For example, as information is added to the master data frame, the columns will contain an alpha code to designate the source of the data (e.g. 'm' for genome scale model or reconstruction, 'u' for UniProt, 'p' for PDB database, etc.). More details are supplied in the Supplementary IPython notebooks and provided master data frames. In this way, we keep track of all of the information that has been mapped from a given database to the GEM. Finally, addressing the quality of the actual structural information will be discussed in more detail below. We approach this task by examining the effects of small-scale sequence variations on individual protein structures, according to the wild-type sequence given by its record obtained through UniProt or other sequence databases.

E. coli

We have used the Blattner gene numbers (b-numbers) present in the *i*JO1366 model for *E. coli* to map the genes to their respective UniProt accession codes (UAC) which then are used to map to protein-related databases. Using a combination of specific identifiers, we achieve maximum coverage of protein structures for a given set of genes in a metabolic model. In the case of the *E. coli* model, we used the blattner (or "b number") identifier as well as the UniProt accession code (UAC) to query the Protein Data Bank (PDB) [1,11]. We specify only reviewed UniProt entries as there is a one-to-one mapping of b-number to UAC for all genes, and only experimental protein structures from the PDB to ensure the highest quality of data in this reconstruction. A further check for available structures was carried out by sequence alignment to the entire PDB, and cross-referenced with the structures identified by the ID mapping and the metadata available in the PDB entry.

Temperature related properties were retrieved by EC number and UniProt accession code through the BRENDA [12] and ProTherm databases [13], respectively. Protein complex information was obtained directly from the EcoCyc database [14] utilizing b-numbers.

T. maritima

We followed a similar procedure for mapping *T. maritima* gene identifiers, with the source of those IDs being the Ensembl Genome database [15]. However, a number of UACs existed as unreviewed entries, and if there was more than one unreviewed entry for a gene, it was manually inspected to ensure the correct mapping. Finally, the available mappings to the PDB database were obtained along with manual alignment to the entire PDB, filtering out of *T. maritima* specific proteins.

Homology Modeling

Fortunately, the increasing number of newly deposited experimental structures provides additional templates for constructing higher quality template-based homology models. In this section, we discuss basic details of the selected homology modeling platform and refer the interested reader to the helpful reviews [16] and methodological reports for more details [17–20]. There are a number of available homology modeling tools and methods to date [4,21–26], many of which perform remarkably well for various types of proteins and conditions [17–20]. Homologous templates are commonly identified

through protein sequence alignments using comparative modeling algorithms [21]. Threading methods [27,28] are capable of identifying common recognizable folds between proteins, even when their evolutionary origins may be different. For query proteins that have no structurally related protein in the PDB, the structure can be built *de novo* through *ab initio* methods [22–24]. Here, we selected the I-TASSER (iterative threading assembly refinement) suite of programs [4,24], which has been the highest ranking program for automated protein structure prediction for the the past two CASP experiments [4,18,26,29].

In the original GEM-PRO models, the homology model for a given gene was selected from a composite of three different homology modeling techniques [30,31]. While this approach proved quite successful, we were interested in updating the choice of homology modeling technique in the updated GEM-PRO models by using a single homology modeling approach for the sake of consistency and reproducibility. We chose a single homology modeling framework, the I-TASSER (iterative threading assembly refinement) suite of programs [4,24] to predict the 3D structures of genes without available experimental structures.

We have filled in the gaps where there are missing structures by querying a previously generated database of I-TASSER homology models for *E. coli* [32,33], and manually generated homology models for all remaining genes in *E. coli* and *T. maritima* [34]. In the final master data frame, we note where available homology models have been mapped to their respective genes. We also include additional information in the data frame that explains the type of computational prediction method used to model the protein structure (e.g. template-based versus *ab initio*), the corresponding URL (for downloading the homology file from the source), the label (i.e. the identifier of the model given by the homology model database), and information related to the confidence of the homology model (e.g. C-score), the native (homologous) template used for the model, etc. All columns added to the master data frame from this stage are preceded by a 'i' for I-TASSER. For most homology modeling procedures, the FASTA (amino acid) sequence of a protein is all that is required to generate a homology model of a protein. It is important to note that certain PDB structures with unresolved residues or gaps in the structure can also be homology modeled to enhance the structural coverage of the amino acid sequence.

We are also interested in assessing the overall quality of the information coming from homologous templates in terms of (i) which organism the protein was crystallized from; (ii) the resolution of the template and (iii) the deposition date. We used these properties to compare the templates that were used to construct homology models in the previous GEM-PRO models with those of the recently updated versions. Using the PDB 4-letter identifier, we first query the PDB database for a numeric taxonomy identifier which we use to query the UniProt taxonomy url (http://www.uniprot.org/taxonomy) for information regarding the organism type. For information regarding the deposition date and the resolution, we use the Python module, ProDy [35].

Finally, we provide comparisons between previous models and their homology models as well as note where there are new templates available for genes that have become available in the last couple years. Comparisons are run utilizing the PSQS program [36] as well as assessing differences in secondary structure, and we also report quality of the current models utilizing the PROCHECK program [37] where

possible. PSQS mainly provides an energy-like measure based on statistical potentials of the mean force between residue pairs, as well as between single residues and solvent. PROCHECK provides geometric checks based on Ramachandran plots. For the current template-based models, a confidence score was assigned to each homology model based on the TM-score, which is a measure that is automatically provided from I-TASSER. A TM-score and root mean squared deviation (RMSD) from the original template provide an estimate that indicates how close the model is to the native structure. In general, TM-score is in the range [0,1] and a value greater than 0.5 indicates higher confidence. The models are also ranked based on the structure density of I-TASSER refinement simulations (for more information, please see [38].

QC/QA Procedure

In some cases, certain genes have more than one crystallographic structure, such as the well-studied structure of lysozyme and its many point mutants. In addition, certain PDB structures may require homology modeling if they are lower in confidence than a standard threshold. Therefore, in the updated GEM-PRO, we provide additional assessments of the quality of all structures (both crystallographic and homology model) mapped to the GEM model. The main objective of this section is to discuss the quality assessment and quality control of the data that has been thus far mapped to the metabolic network reconstruction. In a previous version of GEM-PRO, experimental structures were additionally classified and ranked according to whether a protein was bound to a native metabolite or ligand, in order to ensure proper binding predictions. While the updated version of the GEM-PRO modeling framework does not include the bound state of a protein as a target characteristic in the quality control pipeline, this data is accessible in the knowledge base. Instead, we are mainly interested in quantifying the general quality attributes of the experimental structure of the protein. The final outcome of the quality assessment is the classification of experimental structures into three groups (right panel of Figure 3): (i) high quality structures requiring no modification; (ii) high quality structures requiring minimal (site-directed) modification, and (iii) low quality structures requiring homology modeling. These three metrics ultimately classify PDB files as lower-quality structure if they have a large number (above a set threshold) of point mutations and low SI. For the structures that are flagged as “lower-quality,” we either perform molecular modeling techniques to minimally modify the structure (i.e., if a PDB structure has single point mutations or gaps of less than two sequential residues, which is described in more detail below) or homology modeling.

For structures with multiple chains (that might come from other gene IDs or UACs), we simply rank the PDB file via the alignment score of the chain corresponding to the gene. All sequence alignments were conducted by first extracting the resolved amino acids available in the PDB structure using Biopython and then utilizing the EMBOSS needle package for pairwise sequence alignment between the resolved amino acids and the canonical UniProt sequence [39,40]. In the event that the PDB entry with the best alignment score and the PDB entry with the highest identity do not match, we manually determine whether the structure is suitable or not. The quality scoring metric was designed to assess the quality of,

in terms of completeness and resolution, and rank order all the PDB files for a given gene, based on the following terms,

$$S_{pdb} = S_{SI} + S_{res} + S_{SS} \qquad [S1]$$

where S_{pdb} refers to the total quality score of a single PDB file, which is based on its percent sequence identity (S_{SI}, or coverage of the canonical amino acid sequence), the resolution score (S_{res}) of the crystallographic structure, and, for cases where I-TASSER homology models were available, the similarity (Jaccard similarity) of secondary structural features between the PDB structure and its corresponding homology model (S_{SS}). Furthermore, we also consider the overall completeness of resolved residues in the protein (if there are gaps in resolved amino acids within the structure) and the difference in % α/β composition compared to the I-TASSER homology model. In certain cases, differences in the amino acid sequence between the PDB structure and the homology model (due to insertions, deletions or mutations during crystallography) together with the model refinement generated slight deviations in per-residue secondary structural annotations (see Figure S7). The rankings of all structures for *E. coli* and *T. maritima* are provided in the master data frames (Supplementary Files). Each of the individual metrics are discussed in more detail below. Using a *Z*-score based approach,

$$Z = (X - \mu)/\sigma \qquad [S2]$$

we determine which structures are significantly lower in each of these metrics than the rest of the population. A *p*-value of 0.10 was chosen as a threshold for determining significance ($Z < 1.65$). Structures below a certain cutoff are given lower scores for each metric, and are ranked lower than others with the goal of utilizing the structures for future molecular modeling. However, constructing GEM-PRO models for other organisms can use a less stringent cut-off if fewer crystallographic structures are available. In this case, our methods also allow for a "coarse grained" assessment of mutation type to rank similar mutations (e.g. polar to polar point mutation) higher than dissimilar mutation types (e.g. polar to nonpolar point mutation).

Coverage of the canonical (wild-type) amino acid sequence

The first metric for ranking PDB structures, S_{SI}, scores the sequence identity between what is considered the wild-type sequence (directly from the UniProt, RefSeq, or Ensembl databases) and the PDB sequence [7,8,15,41]. In practice, we use a weighted sequence identity score, which accounts for any bias towards proteins with shorter sequences. Differences in the sequence such as point mutations or amino acids introduced to assist in crystallization of a protein can be found in this way. Higher scoring structures have a better alignment to the wild-type sequence, and cutoffs are set based on the available structures for the genome. As an additional check, we normalize the sequence identity score according to the length of the protein. To do so, the raw percent sequence identity is multiplied by the sequence length, divided by the average total sequence length of the population of PDB structures. This allows for the higher scoring of PDB structures with longer sequence identity overall.

Furthermore, we assess completeness or the degree of missing or unresolved fragments of the protein. This identifies whether there are major sequential gaps in the protein structure, which may require further homology modeling. While S_{SI} will undoubtedly rank structures such as these with lower scores, we are also interested in knowing why a PDB structure has a lower ranking compared to others. This metric is assessed by evaluating observed gaps in the protein (ignoring gaps at the N or C termini) and marking structures with significant gaps as lower quality structures. We have taken the threshold to be one standard deviation from the mean SI as a cutoff for identifying low-quality PDB structures in the model. Similar to the sequence identity, a threshold is determined for each PDB file on the basis of resolution and is reflected in its overall quality score. If an unresolved region of a protein has less than two sequential residues missing, we have carried out standard molecular modeling techniques to minimally modify and insert the missing residue (see the following section, "Structural and sequence refinement" for more details). Otherwise, we perform homology modeling to fill in the larger gaps in sequence. This threshold is chosen based on the confidence in the resulting model: we find that minimal modeling of gaps of two amino acids or less is acceptable using AMBERtools and minimal minimization of the modeled structure. Once a gap in protein structure is greater than two amino acids, we find that I-TASSER suite of programs produces highly confident structures. We also assess whether missing/mutated residues are distributed over the entire protein (i.e., multiple single point mutations) or if they are sequential (i.e., in 'bulk') and where these gaps are located in the protein sequence (we consider all residues that are not within 10 residues from either the N or C termini, due to the inherent flexibility of the protein at these regions).

Resolution quality of the protein structure

The second metric, S_{res}, for ranking PDB structures is based on the resolution, which is a descriptor of the degree of confidence in the resolved atomic coordinates (in Å) of all heavy atoms (for NMR structures, we consider the first member of the ensemble). A higher resolution indicates that a smaller Angstrom distance between atoms can be seen, for instance a structure noted at a 1 Å resolution clearly resolves atoms at or above that distance. The resolution for each structure was obtained from the header section of the PDB file, and cross-referenced with the entry from the PDB website.

Assessment of composition of secondary structural features

The final metric for ranking PDB structures, S_{SS}, assesses the similarity in the percent alpha helix/beta sheet composition of a PDB structure and its matching homology model.

We utilize an implementation of the Jaccard similarity score to measure the similarity of location and length of alpha helices and beta sheets. This metric is only available for structures that have a generated homology model. We reason that if a homology model has been generated utilizing the PDB structure as a template, it has gone through several refinements to become an energetically more favorable structure. This metric allows for comparisons between multiple PDB structures by directly calculating the percentage of secondary structural features.

The difference in per-residue secondary structure is a consequence of the I-TASSER homology modeling procedure but does not indicate that a homology model has lower confidence. For the majority of cases, the homology models in GEM-PRO have been constructed from homologous templates from other proteins with high sequence identities (over 70% sequence identity [42]). In GEM-PRO, missing or mutated parts of the protein have also been homology modeled and refined through the I-TASSER suite

of programs. I-TASSER performs further refinement procedures on the homology model that minimizes the total potential energy of the final structure. Therefore, local (i.e. per residue) changes in secondary structural elements may occur as a result of changing in the secondary amino acid sequence and/or performing the model refinement steps.

While the GEM-PRO quality assessment pipeline has been designed to identify which structures are lower in confidence, we store all available PDB structures for a given gene in the master GEM-PRO data frame. In this way, we ensure that no information is lost in the process of ranking.

Model Refinement

Once the available experimental structures have been compared to the computationally generated homology models, we obtain three sets of structures, some of which may require additional refinement. The first set are experimental structures that meet all criteria above, with an amino acid sequence that matches the canonical “wild-type” mapped from UniProt or other sequence databases. The second set contains structures that differ from the wild-type sequence only by point mutations, and are used as input for this refinement step in order to revert the PDB sequence to the wild-type sequence and fill in missing parts of the protein that do not exceed 1 residue per gap. The third set contains experimental structures that are to be ranked lower than the homology models, due to not meeting the cutoffs as outlined.

The procedure for model refinement is as follows. First, the sequence of amino acids that is resolved in the structure is compared to its mapped sequence. The point mutations are corrected initially using the Biopython structural bioinformatics module [39]. These allow us to change the “mutated” residues to the correct wild-type amino acids by first stripping the R-group atoms, leaving only the peptide backbone atoms of the given residue. Next, the amino acids present in the wild-type sequence are filled in, and the structure PDB file is then passed through the AMBERtools suite of programs (AMBER14) to fill in the heavy atoms of the newly changed amino acid [43]. Homology structures of proteins as well as the models based on crystallographic proteins are modeled at physiological pH. The structure is then minimized with a steepest descent minimization for 10,000 cycles to relieve any overlapping van der Waals interactions. As shown in Figure 5 in the main text, the original crystal structure and the modified structure differ by two residues. The modified structure has been reverted back to the original wild-type sequence. Through the series of modification steps, the final structure aligns exactly with the wild-type sequence and the structure has been minimized to a local minimum. A final QC/QA step was taken by aligning the final wild-type PDB structure to the desired sequence to ensure a final correct structure. In a small handful of cases, the automatic mutation pipeline failed to mutate the correct residue due to inconsistent residue numbering in the PDB file or the use of insertion codes. For these cases, the PDB file was manually altered and minimized.

To summarize, using the above mapping, QC/QA, and refinement pipelines, the updated GEM-PRO models provide representative, high-quality protein structures for a each gene product in the metabolic

model. The overall coverage and quality of the selected experimental and homology-based structures for each organism is detailed in Table 2 of the main text. All mapping-related information has been stored in the GEM-PRO master data frame for each of the model systems.

Model Comparisons

Table S1: Comparisons between previous and current GEM-PRO modeling frameworks. In the comparisons, we evaluate the model coverage in terms of modeling-method-agnostic metrics (sequence identity, coverage of the canonical amino acid sequence), and other properties such as the coverage of protein-metabolite interactions, and protein complex stoichiometry.

**Previous GEM-PRO**	***E. coli***	***T. maritima***
Coverage of genes by at least 1 PDB structure	465/1366	120/478
Coverage of genes by homology models	803/1366	358/478
Maximum sequence coverage	1268/1366	478/478
Coverage of protein-metabolite interactions	24%	--
Coverage of protein complexes	519/1106	--

**Updated GEM-PRO**	***E. coli***	***T. maritima***
Coverage of genes by at least 1 PDB structure	597/1366	149/478
Coverage of genes by homology models	1366/1366	342/478
Number of genes with high quality PDB structures	354/597	112/145
Number of genes with high quality PDB structure requiring point mutations	136/597	24/145
Number of genes with low quality PDB structure to be replaced by homology models	106/597	13/145
Maximum sequence coverage	1366/1366	478/478
Coverage of protein-metabolite interactions	39%	--
Coverage of protein complexes	1085/1106	--

Mapping the PDB to a GEM

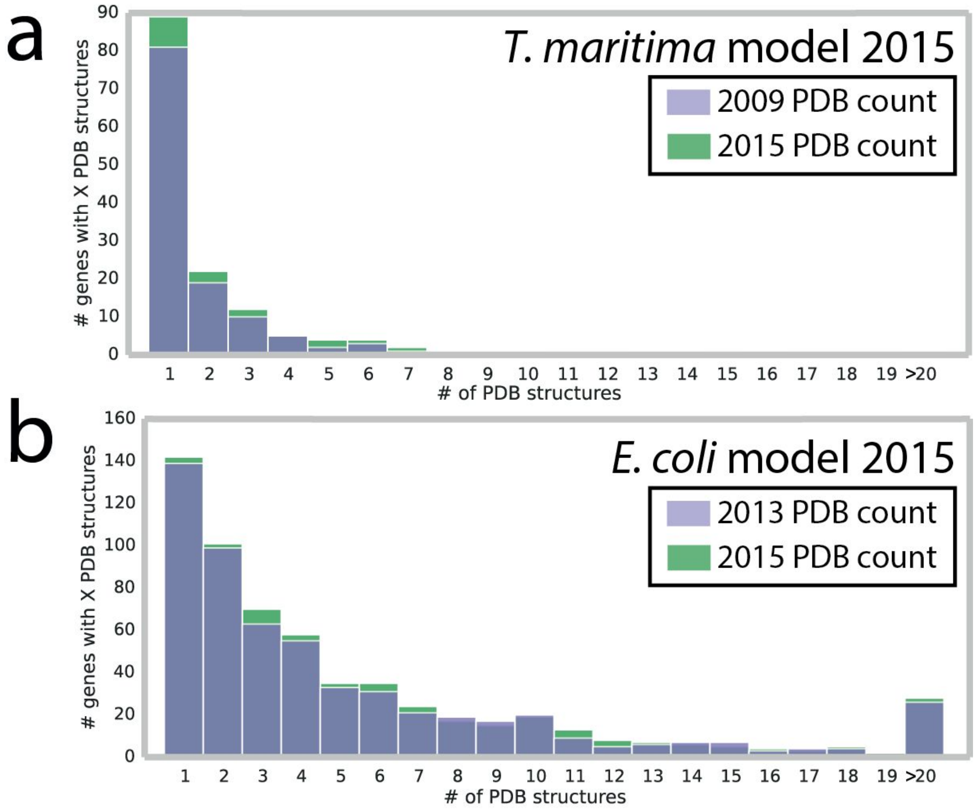

Figure S2: A direct comparison between the old and new GEM-PROs in terms of number of available structures per gene. The distribution of structural coverage of the previously built GEM-PROs (in purple) of (a) *T. maritima,* and (b) *E. coli* are compared to their current 2015 versions (in green).

E. coli

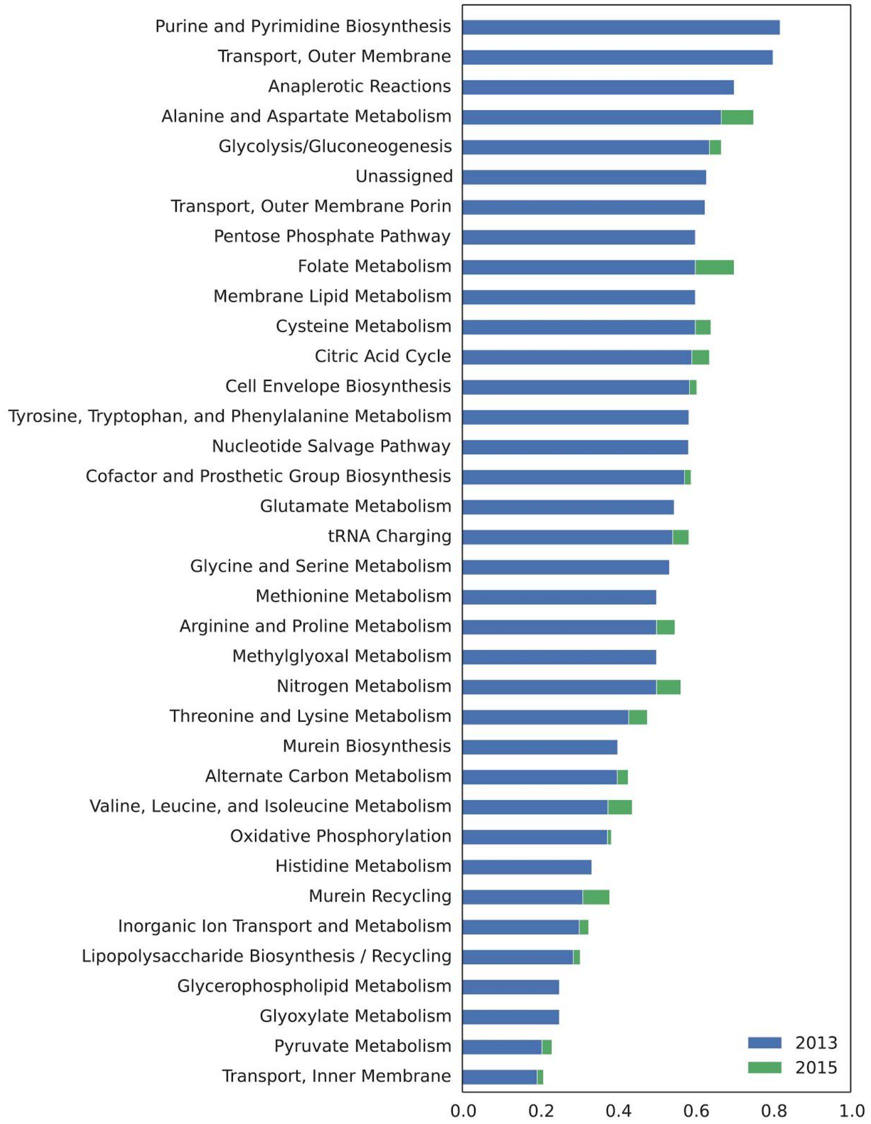

Figure S3: A direct comparison between the original *E. coli* GEM-PRO (2013) and current GEM-PRO indicating which subsystems have newly added crystallographic structures.

T. maritima

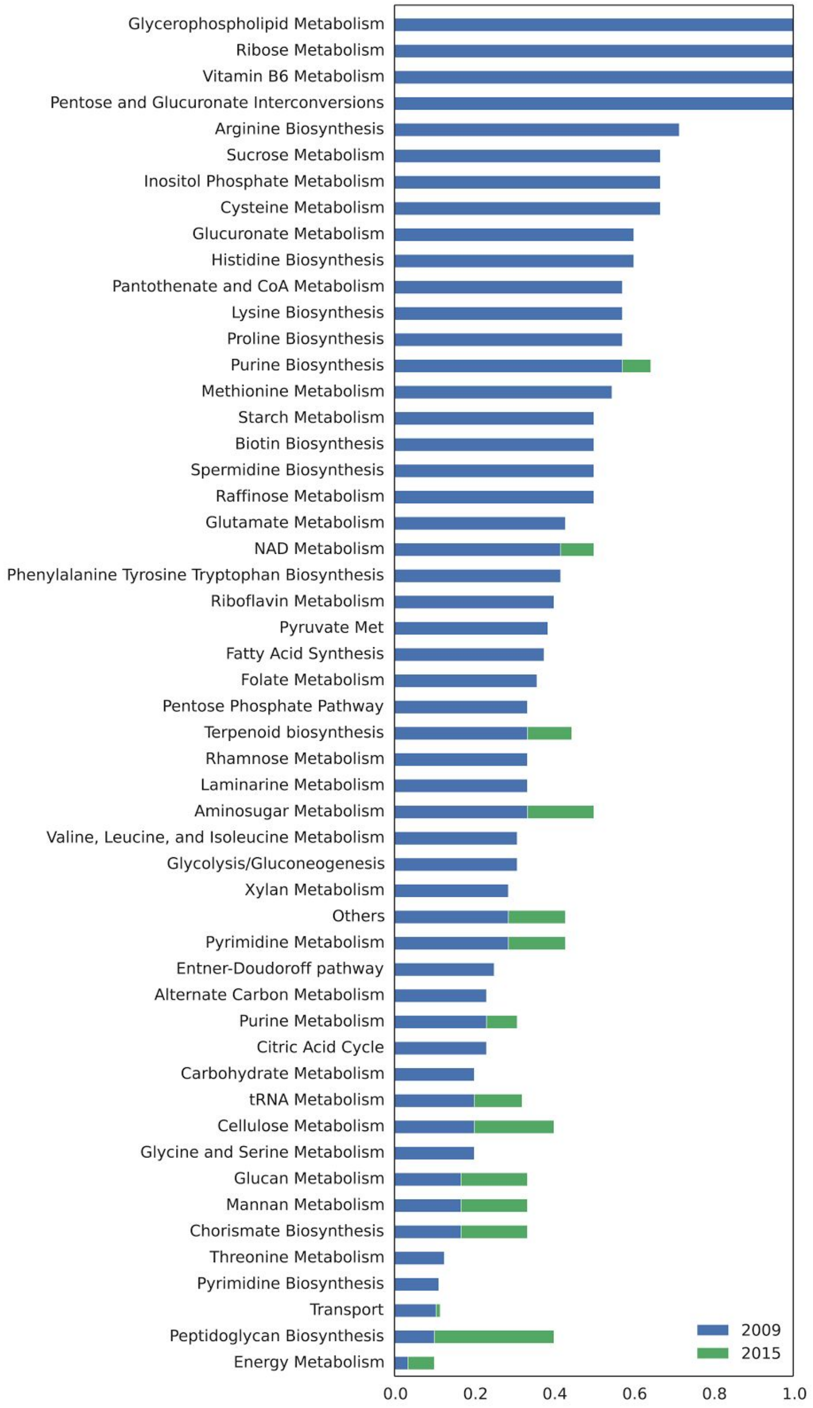

Figure S4: A direct comparison between the original *T. maritima* GEM-PRO (2009) and current GEM-PRO indicating which subsystems have newly added crystallographic structures.

Homology Modeling

The results of the homology modeling pipeline for all three organisms are summarized in Table S2. Where possible, we compare quality information for the new models to compare to the old ones.

Table S2: Structural quality measures for homology models in *T. maritima* and *E. coli*. PSQS provides an total energetic score, with lower scores indicating better quality. PROCHECK provides geometric measures, and a G-factor below -1 is considered unusual. The TM-score is in the range [0,1], with a value >0.5 implying correct topology of a model.

		T. maritima		***E. coli***	
**Method**	**Quality measure**	*2009*	*2015*	*2013*	*2015*
PSQS	Average total score	-0.19 ± 0.10	-0.18 ± 0.11	-0.162 ± 0.10	-0.164 ± 0.12
PROCHECK	Average % of residues in favored positions	-	86.3% ± 5%	88.8% ± 5%	87.1% ± 20%
	Average overall G-factor	-	-0.78 ± 0.2	0.088 ± 0.21	-0.10 ± 0.27
Zhang	TM-score	-	0.79 ± 0.2	-	0.82 ± 0.17

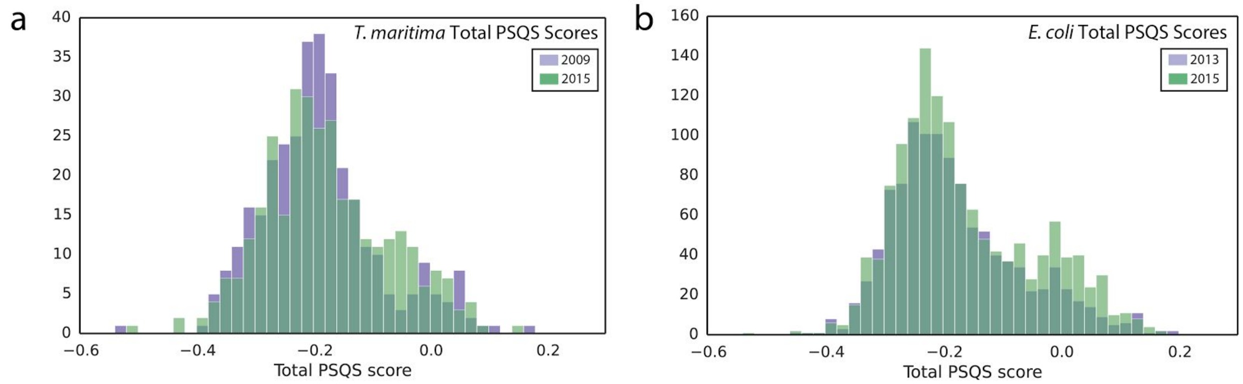

Figure S6: Distribution of total PSQS scores for all homology models in (a) *T. maritima* and (b) *E. coli*. A lower PSQS score indicates higher quality. In (a) and (b), previous distributions of PSQS scores for older GEM-PRO models are shown in purple, while current models are shown in green. In (c), only current PSQS scores are shown as older models were unavailable.

E. coli

Zhang, et al. have previously generated homology models for the *E. coli* genome. 1365 out of the 1366 genes were successfully linked to a homology model in the I-TASSER database. The remaining one gene was modeled utilizing the I-TASSER workflow. Out of the 1366 genes, 23 were modeled *ab initio* without a template, while the remaining 1343 could be modeled with an experimental structure as a template. The complete coverage of the *i*JO1366 model allowed for a detailed comparison between the

models and available PDB structures, discussed in the QC/QA section. The mean TM-score for all homology models in the *E. coli* GEM-PRO is 0.82 ± 0.17.

For the PDB template-based quality checks, we find that the homology models from the previous version of GEM-PRO are derived from 285 different species, with the top 40% of templates coming from *E. coli* (20.5%), *H. sapiens* (7%), *S. typhimurium* (4.8%), *B. subtilis* (3.4%), *T. thermophilus* (3%) and *M. tuberculosis* (3%). The average resolution of all previous templates used is 2.2 ± 0.7 Å. Finally, the most recently deposited templates (73 templates) date back to 2008.

For the recently updated *E. coli* GEM-PRO model, homology models are derived from 183 different species, with the top 44% of templates coming from *E. coli*. The average resolution of all templates used is 2.4 ± 0.7 Å. Additionally, the latest templates being used are from 2012. We find over 50 additional templates since 2008 that are not in the previous model and over 130 newly deposited templates compared to the previous GEM-PRO since 2009.

For the energetic and geometric-based quality checks, we utilize the PSQS and PROCHECK programs in order to assess energetic and conformational stability [36,37]. A negative PSQS score indicates higher quality of a model, while PROCHECK indicates if the conformation of residues are in favored or unfavored positions. find that the average total PSQS score for all homology models in the previous version of GEM-PRO is -0.162 ± 0.10, while the updated models have an average total score of -0.164 ± 0.12 (see Figure S6b for a plot of these scores). The similarities of these two scores can be explained by 1) the updated model now fills in structural gaps (within the protein and also at the termini), potentially basing these regions off of lower quality templates and 2) this is including *ab initio* based models which intrinsically are of lower quality. For current models, PROCHECK reports that the average percentage of residues in favored positions is 87.1% ± 20%, and an average overall G-factor of -0.10 ± 0.27.

T. maritima

For the *T. maritima* model, there were no previously generated I-TASSER models. As a result, we manually generated homology models utilizing the I-TASSER workflow on genes that did not have an experimentally available structure in the PDB. I-TASSER was run utilizing the mapped UniProt amino acid sequences as input, and the results of each run were stored in the final GEM-PRO dataframe. 333 out of the 478 genes in *T. maritima* required the generation of a homology model. All models were generated from a template, with a mean TM-score of 0.79 ± 0.2.

For the PDB template-based quality checks, we find that the homology models from the previous version of GEM-PRO are derived from 102 different species, which is similar to the current *T. maritima* GEM-PRO (103). The top 30% of templates in the previous version are derived from *E. coli* (19%), *T. Thermophilus* (8.3%), *A. Fulgidus* (5.6%) whereas the top 30% of templates from the recent GEM-PRO are derived from *E. coli* (15%), *T. Thermophilus* (7.5%) and *B. subtilis* (3.9%) *T. maritima* (2.7%) and *A. aeolicus* (2.7%). The average resolution for the previous model's templates is 2.2 ± 0.5 Å whereas the average resolution for the updated version is 2.4 ± 0.7 Å. Finally, we find over 100 newly deposited PDB templates (as of 2007) that are now used in the current *T. maritima* GEM-PRO model.

For the energetic and geometric-based quality checks, we find that the average total PSQS score for all homology models in the previous version of GEM-PRO is -0.19 ± 0.10, while the updated models have an average total score of -0.18 ± 0.11 (see Figure S6a for a plot of these scores). These models were generated similarly to the *E. coli* models, and similarly, we are now modeling for gaps to ensure 100% sequence coverage of these models which may account for the slight difference of PSQS score. For current models, PROCHECK reports that the average percentage of residues in favored positions is 86.3% ± 5%, and an average overall G-factor of -0.78 ± 0.2. We were unable to calculate the PROCHECK properties of the older models due to unavailability at the current time.

QC/QA Procedure

E. coli

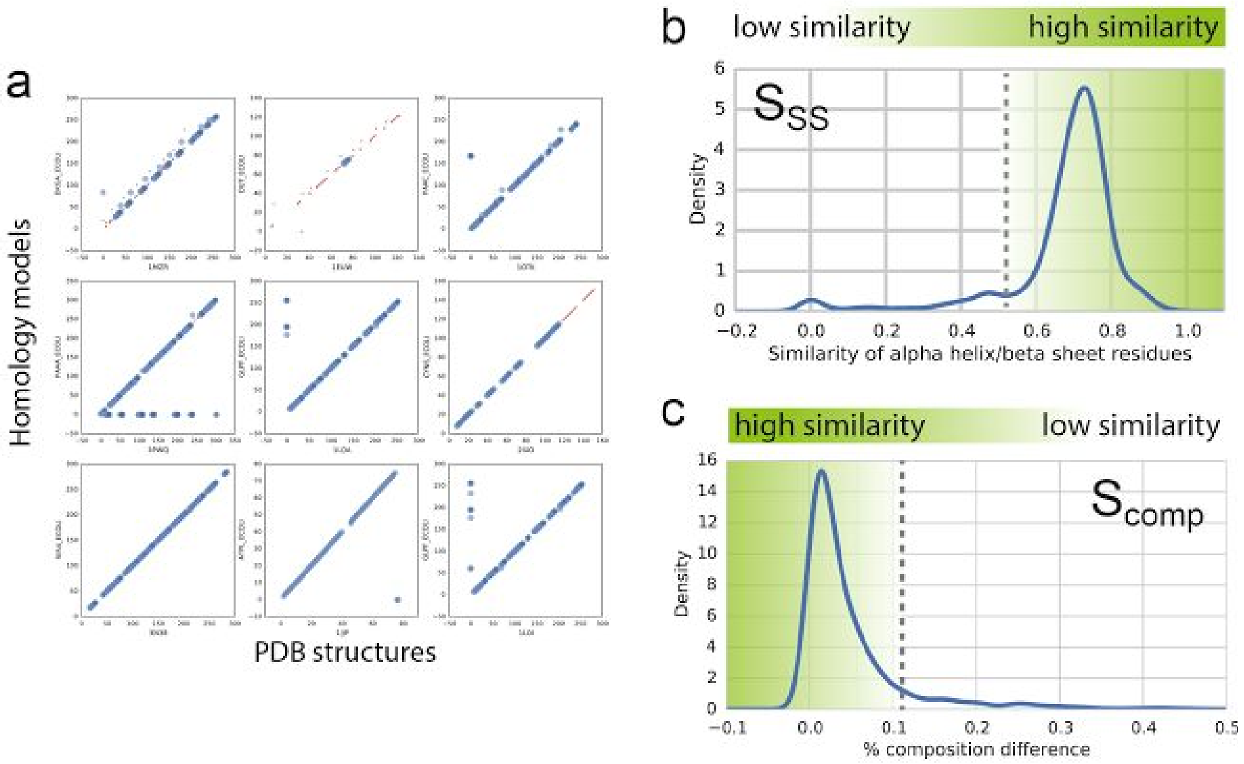

Figure S7: In (a): displayed is a plot of the PDB secondary structure content (alpha helix/beta sheet composition) per residue versus the matching I-TASSER homology model secondary structure content per residue. Blue dots represent alpha helices while red dots represent beta sheets. In (b): The distribution of the Jaccard similarity score in secondary structural content throughout all available PDB-homology pairs. In (c): The distribution of the difference in percent composition of secondary structure content when comparing homology model and experimental structure. In each of the panels, the cut-off threshold is demonstrated by the dashed grey line.

For *E. coli*, the sequence identity cutoff was found to be 83.6%. The lower ranked PDBs are linked to proteins in reactions used for outer membrane transport, tRNA charging and murein biosynthesis

subsystems. Using a resolution cutoff of 2.6 Å filters out structures which are found mostly in the subsystems of outer membrane transport, citric acid cycle and outer membrane porin transport. Finally, the similarity of secondary structure elements cutoff was set at a score of 0.51. Overall, 82% of all available structures are kept as representative structures and subject to further refinement, and 18% are marked to use homology models as the representative structure instead.

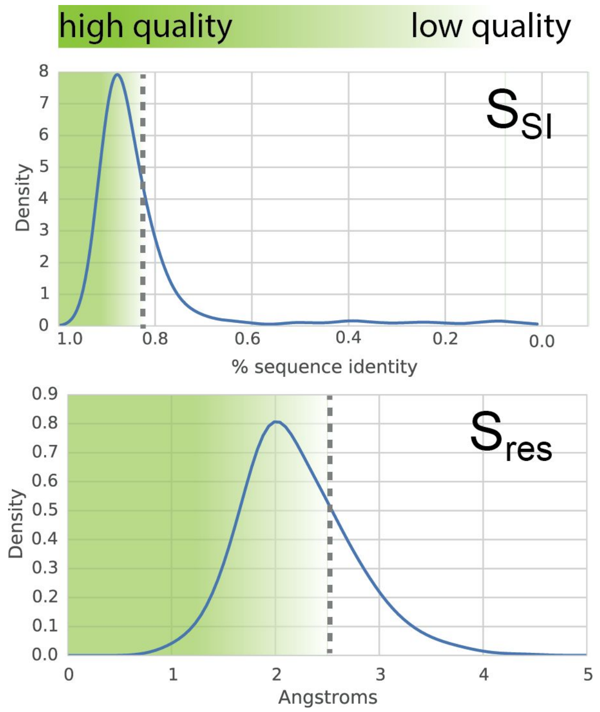

Figure S8: Top: the distribution of the sequence identities for all PDB structures compared to the wild-type *E. coli* sequence across all genes with available crystal structures. Shown by the green filled square, a determined cut-off threshold has been chosen as a means to score the PDB file. Bottom: the distribution of the crystallographic resolution for the PDB structures within the set of genes with available crystal structures. In each of the panels, the cut-off threshold is demonstrated by the dashed grey line.

T. maritima

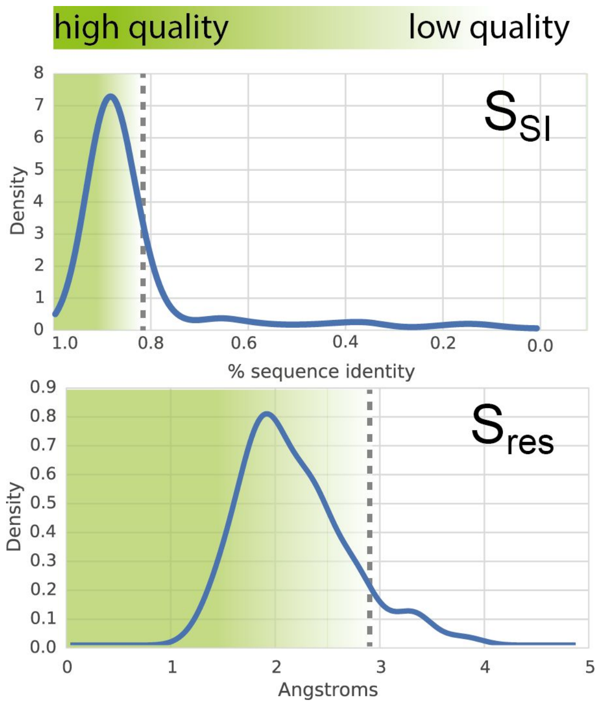

Figure S9: Top: the distribution of the sequence identities for all PDB structures compared to the wild-type *T. maritima* sequence across all genes with available crystal structures. Shown by the green filled square, a determined cut-off threshold has been chosen as a means to score the PDB file. Bottom: the distribution of the crystallographic resolution for the PDB structures within the set of genes with available crystal structures. In each of the panels, the cut-off threshold is demonstrated by the dashed grey line.

For *T. maritima,* the sequence identity cutoff was 82.5%. The lower ranked PDBs are linked to proteins in reactions used for starch metabolism, laminarine metabolism and methionine metabolism. Using a resolution cutoff of 2.9 Å filters out structures which are found mostly in the subsystems of lipid, purine and chorismate biosynthesis. Secondary structure composition similarities and differences were not calculated for *T. maritima* due to the lack of homology models for genes already covered by a PDB structure. Overall, 91% of structures are kept as representative structures and subject to further refinement, and 9% are marked to use homology models as the representative structure instead.

Model Refinement

E. coli

136 experimental structures from the PDB were subject to the model refinement pipeline, and 132 successfully mutated to the correct wild-type sequence. The remaining 4 were manually inspected and had issues mainly due to insertion codes which led to inconsistent numbering in the PDB file. We manually adjusted the content of the PDB file with the correct numbering and reverted the sequence to the wild-type.

T. maritima

24 experimental structures from the PDB were subject to the model refinement pipeline, and 20 successfully mutated to the correct wild-type sequence. The remaining 4 were manually inspected and had numbering issues similar to the *E. coli* structures. These structures were adjusted accordingly in order to provide input to the model refinement pipeline.

Dissemination of GEM-PRO and Development of New Training Resources

Protein Fold Families in Metabolism

A fundamental interest in evolutionary biology is centered on understanding how proteins are organized into the complex biomolecular networks that are observed today. Protein fold family (Pfam) information is a widely used resource for identifying the evolutionary relationship between proteins. Proteins with similar folds or domain organization but little sequence or functional similarity are classified into different "superfamilies" and are assumed to have only a very distant common ancestor. Proteins having the same fold and some degree of similarity in amino acid sequence and/or functionality are classified in "families" and are assumed to have a closer common ancestor. In this section we describe the approach taken to merge protein fold family information together with information related to the metabolic network of *T. maritima* [31] and *E. coli* (see iPython notebook titled, “Protein_Fold_Familes.ipynb”).

In the updated GEM-PRO for *E. coli*, we map a total of 803 unique Pfams across 596 (43%) genes in the metabolic network model, whereas, for *T. maritima*, we find 216 unique Pfams across 143 (29%) genes. In the Supplementary IPython notebook titled, "Protein_Fold_Familes.ipynb" we provide scripts for a basic analysis that enables a comparison of the number of unique folds in proteins across subsystems of metabolism as well as how to find trends in this data. For example, we illustrate the distribution of unique fold families, such as the Rossmann fold domain, across all metabolic subsystems. The Rossmann fold is highly distributed across various genes in several specific subsystems in both *T. maritima* and *E. coli*, which include cofactor biosynthesis (16), cell envelope biosynthesis (9), and oxidative phosphorylation and TCA cycle (8). Previously, Pfam and SCOP classification information were used in conjunction with a genome-scale modeling approach for the discovery and characterization of evolutionary structural folds and domains throughout metabolism [31]. This study has enabled a comparison of “patchwork” [44,45] and “retrograde” [46] models of enzyme evolution.

E. coli

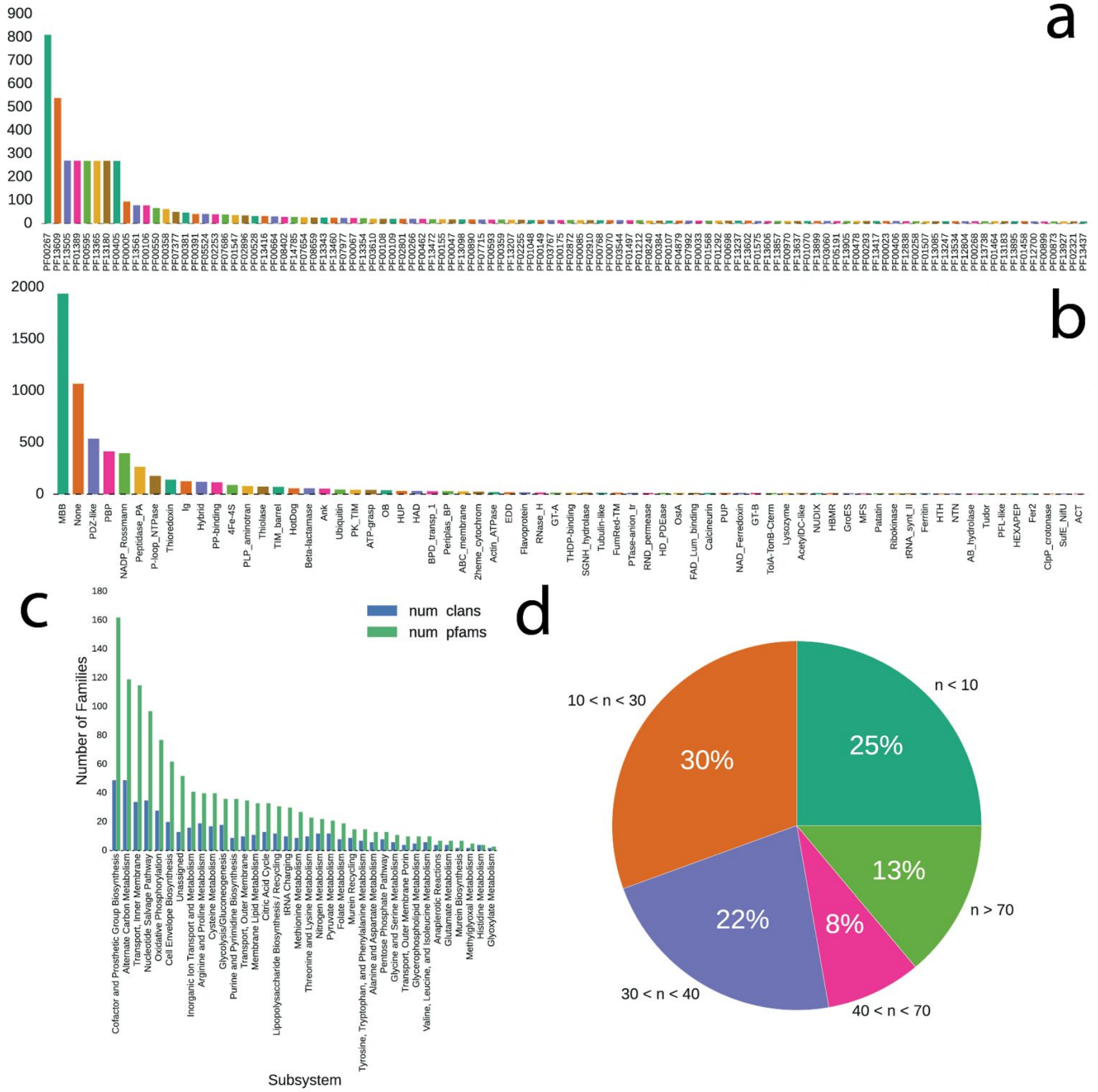

Figure S11: Coverage of protein fold families in *E. coli* GEM-PRO model. In (a), a bar plot indicating the distribution of PFAM accession numbers (x-axis) across different genes in the metabolic model. In (b), the distribution of clans across all genes in the metabolic network. In (c) is the distribution of clans and PFAM accession numbers across subsystems in the metabolic network. In (d) is a pie chart that presents the number of subsystems in *E. coli* with *n* number of PFAM types. For example, half of all subsystems have less than 30 unique PFAMs and one quarter have less than 10.

m_subsystem	norm_clan	norm_pfam	num_clans	num_genes	num_pfams
Transport, Outer Membrane Porin	1.4	0.636364	5	7	11
Transport, Inner Membrane	5.904762	1.467456	42	248	169
Transport, Outer Membrane	2.181818	0.648649	11	24	37
Nucleotide Salvage Pathway	1.813953	0.661017	43	78	118
Glycerophospholipid Metabolism	2.4	1.090909	10	24	22
Alternate Carbon Metabolism	2.78125	1.011364	64	178	176
Cofactor and Prosthetic Group Biosynthesis	3.086207	0.832558	58	179	215
Cell Envelope Biosynthesis	1.962963	0.654321	27	53	81
Murein Recycling	2.416667	1.16	12	29	25
Nitrogen Metabolism	1	0.533333	16	16	30
Arginine and Proline Metabolism	1.863636	0.803922	22	41	51
Membrane Lipid Metabolism	1.071429	0.384615	14	15	39
Pyruvate Metabolism	2.294118	1.114286	17	39	35
rosine, Tryptophan, and Phenylalanine Metabo	2.4	1.142857	10	24	21
Valine, Leucine, and Isoleucine Metabolism	1.666667	1	9	15	15
Lipopolysaccharide Biosynthesis / Recycling	2.24	1	25	56	56
Unassigned	2.428571	0.586207	14	34	58
Citric Acid Cycle	1.466667	0.536585	15	22	41
Cysteine Metabolism	1.315789	0.555556	19	25	45
Purine and Pyrimidine Biosynthesis	2	0.55	11	22	40
Inorganic Ion Transport and Metabolism	3.28	1.171429	25	82	70
Methionine Metabolism	1.071429	0.441176	14	15	34
Alanine and Aspartate Metabolism	1.714286	0.857143	7	12	14
tRNA Charging	1.846154	0.615385	13	24	39
Methylglyoxal Metabolism	2.666667	1.333333	3	8	6
Threonine and Lysine Metabolism	1.818182	0.689655	11	20	29
Histidine Metabolism	0.9	0.818182	10	9	11
Oxidative Phosphorylation	2.75	0.933962	36	99	106
Glycine and Serine Metabolism	2.142857	1	7	15	15
Pentose Phosphate Pathway	1.666667	0.882353	9	15	17
Glycolysis/Gluconeogenesis	1.65	0.825	20	33	40
Folate Metabolism	1	0.454545	10	10	22
Glutamate Metabolism	1.375	0.785714	8	11	14
Glyoxylate Metabolism	1	0.8	4	4	5
Anaplerotic Reactions	2	1	5	10	10
Murein Biosynthesis	2	0.909091	5	10	11

Table S3: Displayed are the various subsystems in *E. coli* metabolism and the number of pfams (num_pfams), number of clans corresponding to those pfams (num_clans), these values normalized to the number of genes (num_genes) in the particular subsystem (norm_pfams and norm_clans, respectively).

m_subsystem	norm_clan	norm_pfam	num_clans	num_genes	num_pfams
Cysteine Metabolism	1.2	0.666667	5	6	9
Pyruvate Met	2.166667	1.3	6	13	10
Fatty Acid Synthesis	2	0.888889	4	8	9
Arginine Biosynthesis	1.166667	0.875	6	7	8
Valine, Leucine, and Isoleucine Metabolism	2.4	1.5	5	12	8
Citric Acid Cycle	2.6	2.6	5	13	5
Pantothenate and CoA Biosynthesis	1	1	1	1	1
Lysine Biosynthesis	1.4	1	5	7	7
Purine Metabolism	2.6	1.3	5	13	10
Methionine Metabolism	1.833333	1	6	11	11
Purine Biosynthesis	2.333333	0.666667	6	14	21
Aminosugar Metabolism	1.5	1.2	4	6	5
Lipid Biosynthesis	2	2	2	4	2
Thiamine Biosynthesis	3	3	1	3	1
Folate Metabolism	2.8	1.4	5	14	10
Peptidoglycan Biosynthesis	3.333333	2.5	3	10	4
tRNA Metabolism	4.166667	2.777778	6	25	9
Transport	11.875	5.277778	8	95	18
Alternate Carbon Metabolism	2.6	1.444444	5	13	9
Others	1.5	1	4	6	6
Starch Metabolism	0.5	0.5	4	2	4
Phenylalanine Tyrosine Tryptophan Biosynthesis	4	1.5	3	12	8
Riboflavin Metabolism	1	1	5	5	5
Arabinose Metabolism	2	2	1	2	1
Threonine Metabolism	4	1.333333	2	8	6
Alanine and Aspartate Metabolism	3	3	1	3	1
Pyrimidine Biosynthesis	4.5	4.5	2	9	2
NAD Metabolism	1.714286	1.333333	7	12	9
Glutamate Metabolism	1.75	0.875	4	7	8
Histidine Biosynthesis	1.428571	1	7	10	10
Energy Metabolism	6	3.75	5	30	8
Biotin Biosynthesis	1	0.666667	2	2	3
Carbohydrate Metabolism	3.333333	3.333333	3	10	3
Cellulose Metabolism	1.25	1.25	4	5	4
Butanoate Metabolism	2	2	1	2	1

Terpenoid biosynthesis	2.25	1.285714	4	9	7
Chorismate Biosynthesis	1.5	1.2	4	6	5
Pyrimidine Metabolism	2.8	1.75	5	14	8
Glucuronate Metabolism	1.25	1.25	4	5	4
Entner-Doudoroff pathway	2	2	2	4	2
Pantothenate and CoA Metabolism	1.75	0.875	4	7	8
Ribose Metabolism	1	0.5	1	1	2
Proline Biosynthesis	2	0.75	3	6	8
Glycolysis/Gluconeogenesis	1.857143	1.857143	7	13	7
Pentose Phosphate Pathway	2.25	2.25	4	9	4
Galactose metabolism	7	7	1	7	1
Mannan Metabolism	3	3	2	6	2
Raffinose Metabolism	0.666667	0.666667	3	2	3
Glycine and Serine Metabolism	2.5	0.833333	2	5	6
Glycogen synthesis	3	3	1	3	1
Xylan Metabolism	2.333333	2.333333	3	7	3
Glucan Metabolism	2	1.2	3	6	5
Glycogen Metabolism	1	1	1	1	1
Serine Metabolism	1	1	1	1	1
Glycerophospholipid Metabolism	1	1	1	1	1
Pentose and Glucuronate Interconversions	1	1	1	1	1
Inositol Phosphate Metabolism	0.75	0.75	4	3	4
Laminarine Metabolism	1	0.75	3	3	4
Maltose Metabolism	0.666667	0.666667	3	2	3
Mannitol Metabolism	1	1	1	1	1
Spermidine Biosynthesis	1	0.666667	2	2	3
Vitamin B6 Metabolism	1	0.333333	2	2	6
Rhamnose Metabolism	1.5	1.5	2	3	2
Sucrose Metabolism	0.6	0.6	5	3	5
Fructose Metabolism	1	1	1	1	1

Table S4: Displayed are the various subsystems in *T. maritima* metabolism and the number of pfams (num_pfams), number of clans corresponding to those pfams (num_clans), these values normalized to the number of genes (num_genes) in the particular subsystem (norm_pfams and norm_clans, respectively).

We compared the spread of molecular motifs by comparing the distribution of annotated Pfams across metabolism. For *E. coli*, we map a total of 1159 unique Pfam folds to gene products in GEM-PRO (596 of which are taken from the official Pfam database and 775 have been generated from the publicly available source code [47]). Our findings suggest that there are a total of 1162 unique Pfams in *E. coli* metabolism, suggesting that a great deal of proteins share the same motif. For *T. maritima*, we identified 143 (29%) genes with Pfam annotations and 147 genes with predicted Pfam annotations. As the total

number of genes in the metabolic model for *T. maritima* is much lower than for *E. coli* (478 versus 1366, respectively), it is not surprising that the number of unique Pfams is also lower in *T. maritima* (216). Of these Pfams, many are readily distributed across a range of metabolic subsystems, such as glycolysis, citric acid cycle, alternative carbon metabolism and cofactor and prosthetic group biosynthesis (See Figure S11 (a-c)).

Classifying Pfams into clans allows for identifying functional motifs, such as ligand or cofactor binding domains. In *E. coli*, we identify 175 unique clans that represent the distinctive molecular features in all Pfams in metabolism. One example of the most predominant clan is that of the Rossmann fold, or NADP-binding motif. In contrast, other motifs, such as the FAD oxidoreductase-C terminal domain, are observed in only three subsystems and in two different genes, namely glcD, dld. Despite the difference in gene count between *E. coli* and *T. maritima*, the average number of unique clans per subsystem for both organisms 2.1. Further, the subsystems with the maximum number of unique clans in both organisms are related to inner and outer membrane transport, and those with the fewest number of unique clans are histidine biosynthesis in *E. coli* and arginine and cysteine biosynthesis in *T. maritima.*

Growth at Different Temperatures

Thermotolerance and thermosensitivity are protein properties that determine the catalytic activity of proteins at various temperatures. Once a knowledgebase of temperature-related data is collated and mapped to a metabolic network model of a specific organism, the growth rate can be predicted across a range of temperatures. Furthermore, both *in silico* and *in vivo* experiments have been designed to pinpoint which reactions in the metabolic network of *E. coli* are rate-limiting at a given temperature [30]. In this section, we provide an example as a tutorial of a previously published approach to predict temperature-dependent growth rate of *E. coli*. To illustrate this previous application of GEM-PRO (*i*RC1366-GP), the predicted and measured temperature data together with the methodology of integrating this data with constraint-based methods is provided as a Supplementary IPython notebook, titled "Temperature_Dependent_Growth_ Prediction.ipynb."

Different temperature-related metrics, including the critical temperature of cold denaturation, freezing point, optimal temperature, melting point, and temperature of heat denaturation are queried from online databases or predicted using previously published methods [48–51], and integrated into the GEM-PRO model of *E. coli*. Using a constraint-based modeling approach, we use the temperature data to add further constraints to the genome-scale model of *E. coli* (*i*JO1366) to simulate growth under different temperatures. The maximum flux through a given reaction was set by a temperature-based activity function, which considers the molar fraction of protein in the native folded state (versus the denatured state) and a minimum biomass requirement. Using this approach, both the growth rate of *E. coli* at different temperatures as well as the proteins predicted to participate in the rate-limiting reactions (at a given temperature) were predicted and found to be in good agreement with experimentally measured growth in matching conditions [30].

E. coli

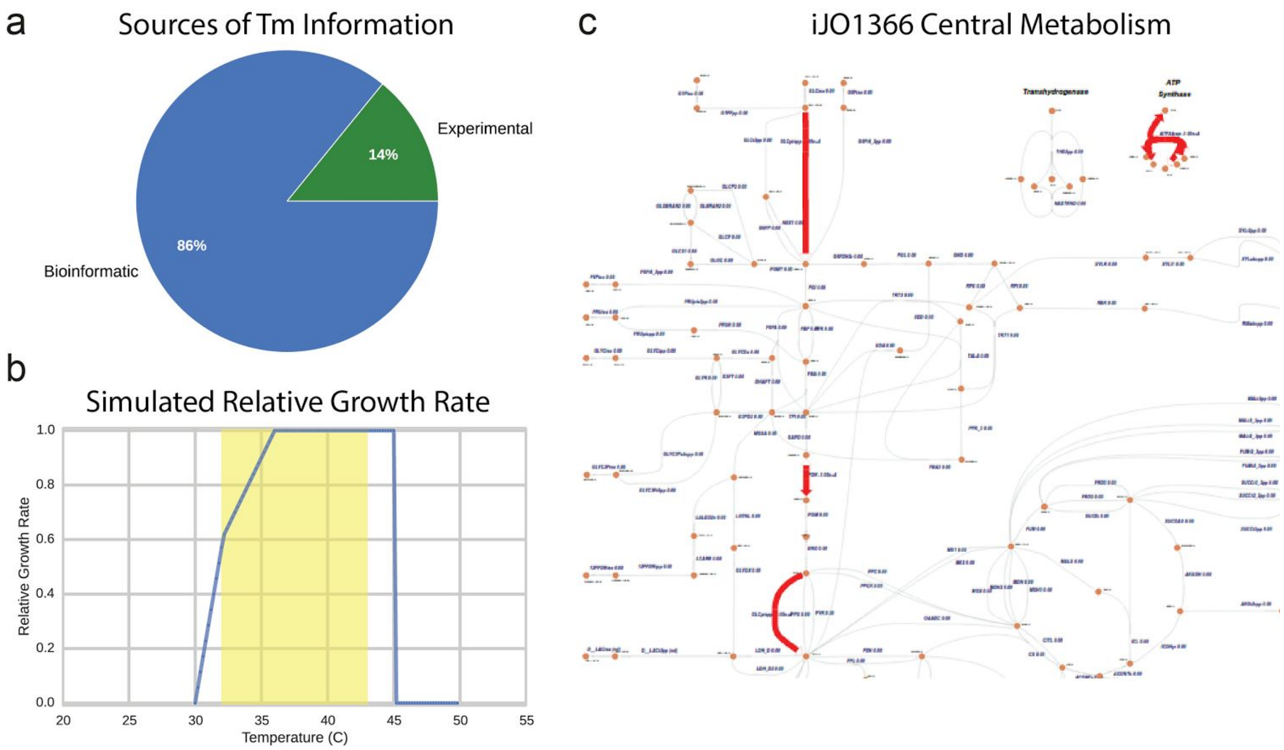

Figure S12: (a) Sources of predicted melting temperature values coming from different *in silico* or experimental methods. (b) Simulated growth of *i*JO1366 with temperature constraints added. Highlighted in yellow is the region that correlates well with experimental growth rates per Chang et al., 2013. (c) Reaction “hot spots”, or pathways of the metabolic pathway that are limited due to the temperature constraints added to a simulation at 42.2 ℃.

Metabolite Versatility in co-crystallized complexes

E. coli

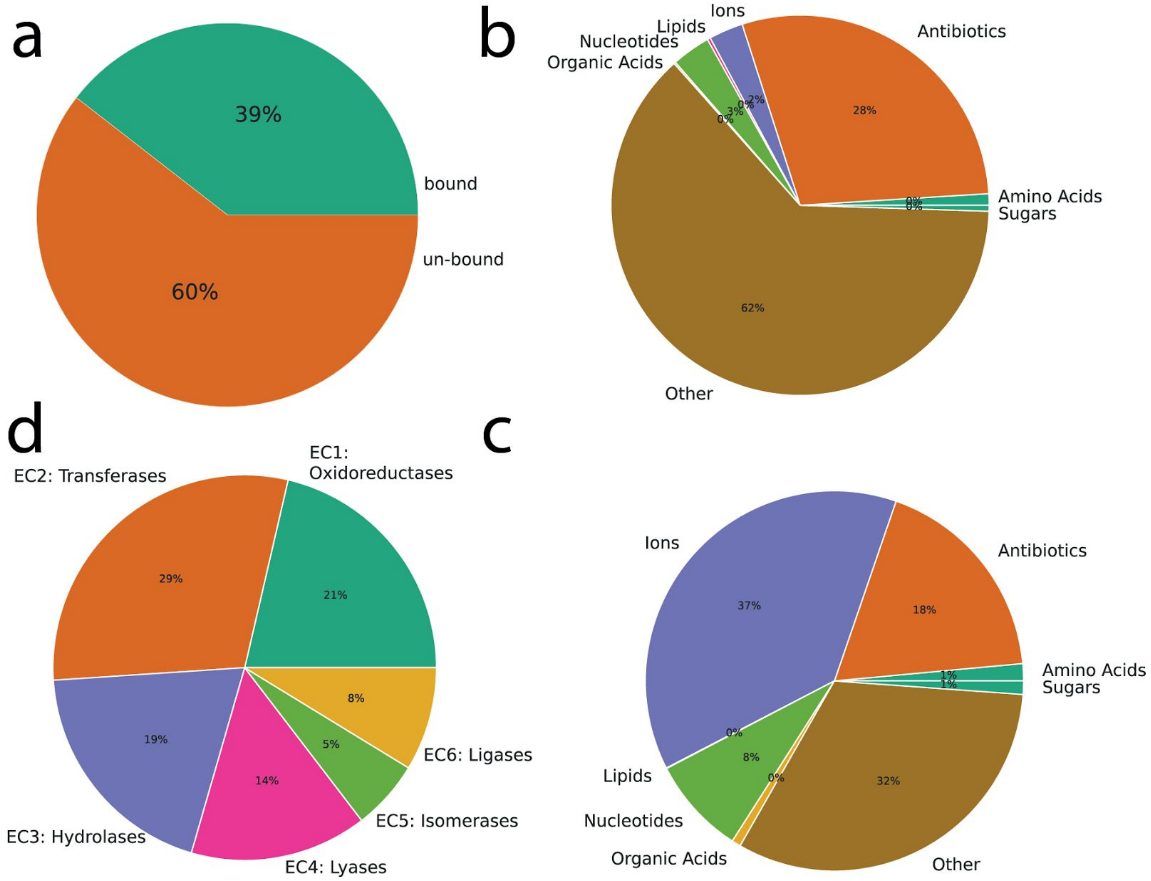

Figure S13: In (a) is the total number of crystallographic structures in *i*JO1366 that are co-crystallized to substrates (bound) or apoenzymes (unbound). In (b), the total number of all ligands in the PDB ligand expo database were classified based on a previous approach [52] into various types of ligands. In (c), the same classification scheme was carried out on *i*JO1366 GEM-PRO ligands. In (d) are the various reaction types in *i*JO1366 (given by their EC number) and how many are co-crystallized with ligands or substrates.

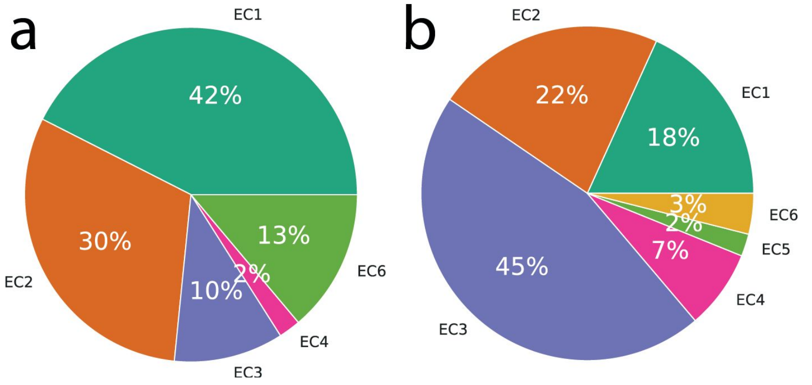

Figure S14: In (a) is the minimum variance (top 10% of most similar ligands bound to genes in the *E. coli* model) in substrate similarity (determined via Tanimoto coefficients of InChI metabolite footprint similarities) between all reactions in iJO1366,

classified by EC number and in (b) is the maximum variance (top 10% of most diverse ligands bound to genes in the *E. coli* model).

Enzyme Abundances and Protein Complex Stoichiometry

E. coli

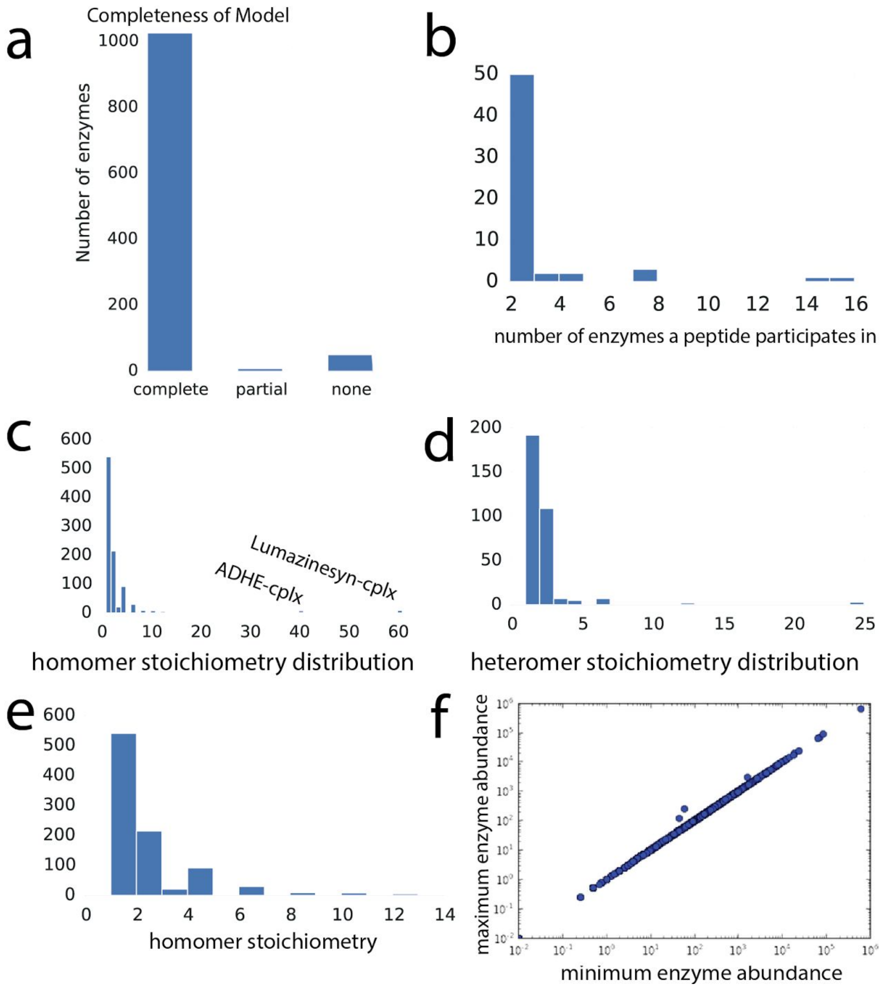

Figure S15: In (a) displayed is the coverage of enzyme complex stoichiometry for the *E. coli* metabolic model. In (b), is the distribution of the number of enzyme complexes a peptide participates in. In (c) and (d) are homomer and heteromer stoichiometry distributions. In (e), a zoom of the homomer stoichiometry graph in (c) shows a preference for even stoichiometry. In (f), using complex stoichiometry coupled with ribosome profiling data and constraint-based modeling, the maximal and minimal abundances are computed using flux variability analysis assuming free peptide abundance are minimized. As shown in the plot, the range of potential enzyme abundance indicates that enzyme abundances are quite constrained by stoichiometry. While GEM-PRO focuses on proteins in metabolism, curation efforts have also led to the reconstruction of stoichiometry for enzymes involved in protein synthesis, which is available in the original publication [53]. The updated version of GEM-PRO for *E. coli* reports 1034 enzyme complexes with their respective complex stoichiometries, which is 500 additional enzyme complexes more than the previous version of the GEM-PRO (iRC1366-GP) [54]. Of the 1034 complexes, 574 can be mapped to experimental structures and the remaining can be mapped to homology models. For *T. maritima*, 27% of the genes have complete or partial structural coverage of one or more of the subunits in the enzyme complex.

Proteins with the largest free abundances

- Aspartate carbamoyltransferase regulatory chain
- Acetyl-CoA carboxylase carboxyltransferase subunit beta {ECO:0000255|HAMAP-Rule:MF_01395}
- ACCase subunit beta {ECO:0000255|HAMAP-Rule:MF_01395}
- Acetyl-coenzyme A carboxylase carboxyl transferase subunit beta {ECO:0000255|HAMAP-Rule:MF_01395}
- Dipeptide transport ATP-binding protein DppD
- Dipeptide transport ATP-binding protein DppF
- Arginine transport ATP-binding protein ArtP
- ABC transporter arginine-binding protein 1
- Putative ABC transporter arginine-binding protein 2
- PTS-dependent dihydroxyacetone kinase, ADP-binding subunit DhaL
- Thiosulfate-binding protein
- Sulfate transport system permease protein CysT

Comparative Systems Biology of Different Species

The homologs of *T. maritima* and *E. coli* were found using a bi-directional (BBH) best hit BLASTP search of genomes for *E. coli* K-12 MG1655 and *T. Maritima* MSB8. The BBH search was performed using annotated genomes from the RAST server [55] with a minimum cutoff of 20% sequence identity. We compared structural similarity between *E. coli* and *T. maritima* homologs as well as an all-versus-all comparison, using a pairwise structural alignments using the FATCAT algorithm [56].

E. coli proteins that share structural similarity with the most *T. maritima* proteins are involved in reactions such as succinyl-CoA synthetase (ADP-forming) (60), pyruvate kinase (42), phosphogluconate dehydrogenase (39), ribulose 5-phosphate 3-epimerase (33). Of this highly similar subset of proteins, only half of the cases shared one or two Pfam domains and a slight majority (56%) share at least one metabolite in common. Secondary carbon metabolism reactions, including Fructoselysine kinase and glutamyl-tRNA reductase, had relatively fewer structural matches (6 and 7 respectively), in *T. maritima* subsystems such as terpenoid biosynthesis, pantothenate and CoA metabolism, peptidoglycan biosynthesis, folate metabolism and others. All alignments were clustered based on the root-mean-squared-deviation (RMSD) of the protein backbones and considered only alignments with coverages of greater than 70% of the protein. Alignments with high overlap were considered to have an RMSD less than 5 Å. In central carbon metabolism, we identified proteins in both the *E. coli and T. maritima* metabolic networks that have highly similar structural domains to a large set of proteins, and are involved in reactions such as transaldolase, fructose-bisphosphatase, isocitrate dehydrogenase (NADP-dependent), glucose-6-phosphate isomerase, malate dehydrogenase and citrate synthase.

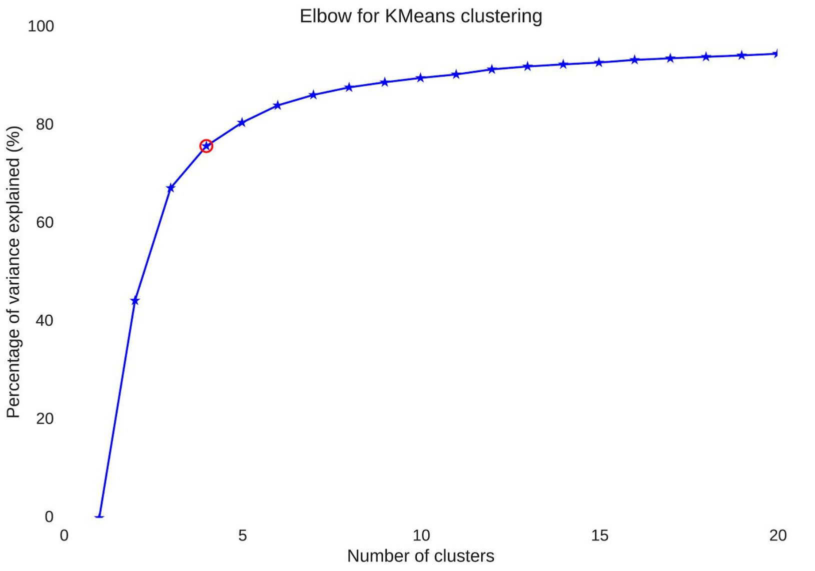

Figure S16: Explained Variance. Here we look at the percentage of variance explained as a function of the number of clusters from K-means clustering. We are interested in choosing the number of clusters such that adding another cluster doesn't give a largely different coverage of the data (i.e. the marginal gain drops off). At the point in the graph where the marginal gain drops, we find an angle (or "elbow") in the graph. The number of clusters is chosen at this point, hence the "elbow criterion". The "elbow" is indicated by the red circle and the number of clusters chosen was 4.

To calculate the different the different physico-chemical properties, we took all crystallographic structures and homology models in the GEM-PRO models. For the crystallographic structures, we filtered out all chains not corresponding to the gene of interest, such that each structure file consisted of a single monomeric chain. The chain corresponding to the gene of interest was found via the 'p_uniprot_chain_map' column in the GEM-PRO master dataframe, which provides a mapping of the chains in the PDB file to the uniprot accession number, which can then be linked to the gene of interest. We then evaluated the computed properties of the entire proteomes of *E. coli* and *T. maritima* individually as well as combined, using PCA and K-means clustering algorithms (see below).

Multivariate analyses, like principal component analysis (PCA), allow for the identification of correlation in different protein structural properties, which may be correlated based on a given metabolic subsystem, across catalytic reaction types, or another grouping. To carry out such an analysis, all data was organized into a matrix where each column represents the values of a different property, (such as solvent-exposed surface area, or SASA), and each row is a given protein. Each of the above properties was normalized by the range of values of the specified property across all proteins. The eigenvalues, λ_i, of the matrix represent the variance of a property (or the degree of correlated change in the data set) which is associated with each axis that is formed as a result of performing PCA. The coefficients of an eigenvector indicate the contribution of the original variables to the vector and are referred to as factor loadings. When the variance is small along a number of axes, it can be ignored and, thus, a reduction of the original highly dimensional data is achieved. For statistical analyses, like PCA and K-means clustering, we used the sklearn [57] and scipy [58] python packages.

Comparing 29 different physical properties (see Table S5), we find that the largest differences in mean-normalized values between *E. coli* and *T. maritima* are associated with relative SASA (average per residue solvent accessibility per protein), the percent of nonpolar residues on the surface of the protein, percent of polar residues on the surface of the protein, percent of positive residues on the surface of the protein, percent of negative residues on the surface of the protein, and percent of buried nonpolar residues. In particular, we find there to be an increase in buried nonpolar residues in *T. maritima* relative to *E. coli*. In *T. maritima*, we also find there to be significant depletion in solvent accessibility and polar and nonpolar surface residues and significant enrichment in charged surface residues and buried nonpolar residues compared to *E. coli*. Some of the most interesting differences between *E. coli* and *T. maritima* are related to relative SASA, which indicate that proteins in the thermophilic metabolic network are on average larger (and less solvent accessible) than proteins in *E. coli* metabolism. In the largest cluster of proteins, we find an increase in the number of proteins with surface charges and solvent accessibility and a depletion in polar residues, while the second group of proteins has an enrichment in polar residues and is depleted in the surface charge and average solvent accessibility. The transmembrane proteins found in the first cluster (e.g., porins) are mainly correlated because their surfaces are made of mostly occupied by α-helices with much lower protein surface charge compared to that of the second cluster.

Based on the clustering analysis described above, we also find that the majority (61%) of all *E. coli* “hotspot” proteins are in cluster 3 and 23% are in cluster 2. While cluster 3 happens to be the largest cluster (41% of all *T. maritima* and *E. coli* proteins), the majority of proteins in this cluster have increased solvent-exposed surface areas, which is likely to make them less stable at higher temperatures.

**GEM-PRO descriptor**	**Description**
ovality	(SASA/Nres^2/3)
ssb_avg_res_depth	average angstrom distance to surface<br>(averaged over all atoms in residue)
ssb_ca_depth	average angstrom distance to surface<br>(averaged over all alpha carbon atoms in residue)
ssb_cys_bridge	number of cysteine bridges
ssb_mean_rel_exposed	relative mean exposed surface area
ssb_per_310_helix	percent 310 helical residues in protein
ssb_per_5_helix	percent pi helical residues in protein
ssb_per_B	percent of buried residues in protein
ssb_per_B_NP	percent of buried nonpolar residues in protein
ssb_per_B_P	percent of buried polar residues in protein
ssb_per_B_neg	percent of buried negative residues in protein
ssb_per_B_pos	percent of buried positive residues in protein
ssb_per_NP	percent nonpolar residues in protein
ssb_per_P	percent polar residues in protein
ssb_per_S	percent of surface residues in protein

ssb_per_S_NP	percent of surface nonpolar residues in protein
ssb_per_S_P	percent of surface polar residues in protein
ssb_per_S_neg	percent of surface negative residues in protein
ssb_per_S_pos	percent of surface positive residues in protein
ssb_per_alpha	percent alpha helical residues in protein
ssb_per_bent	percent bent/coil residues in protein
ssb_per_beta_bridge	percent beta bridge residues in protein
ssb_per_ext_beta	percent beta sheet residues in protein
ssb_per_hbond_turn	percent residues participating in hydrogen bonds at turns in protein
ssb_per_irr	percent disordered residues in protein
ssb_per_neg	percent negative residues in protein
ssb_per_pos	percent positive residues in protein
ssb_sasa	solvent exposed surface area
ssb_size	total size (amino acid length)

Table S5: The GEM-PRO columns corresponding to computed structural properties and their descriptions.

References

1. Berman HM, Westbrook J, Feng Z, Gilliland G, Bhat TN, Weissig H, et al. The protein data bank. Nucleic Acids Res. Oxford Univ Press; 2000;28: 235–242.

2. Lewis NE, Nagarajan H, Palsson BO. Constraining the metabolic genotype--phenotype relationship using a phylogeny of in silico methods. Nat Rev Microbiol. Nature Publishing Group; 2012;10: 291–305.

3. Ebrahim A, Lerman JA, Palsson BO, Hyduke DR. COBRApy: COnstraints-Based Reconstruction and Analysis for Python. BMC Syst Biol. 2013;7: 74.

4. Zhang Y. I-TASSER: Fully automated protein structure prediction in CASP8. Proteins: Struct Funct Bioinf. Wiley Online Library; 2009;77: 100–113.

5. Hucka M, Finney A, Sauro HM, Bolouri H, Doyle JC, Kitano H, et al. The systems biology markup language (SBML): a medium for representation and exchange of biochemical network models. Bioinformatics. 2003;19: 524–531.

6. Schellenberger J, Park JO, Conrad TM, Palsson BØ. BiGG: a Biochemical Genetic and Genomic knowledgebase of large scale metabolic reconstructions. BMC Bioinformatics. 2010;11: 213.

7. Magrane M, Consortium U. UniProt Knowledgebase: a hub of integrated protein data. Database . 2011;2011: bar009.

8. Berman HM. The Protein Data Bank. Nucleic Acids Res. 2000;28: 235–242.

9. McKinney W. Data structures for statistical computing in Python. Proceedings of the 9th Python in Science Conference. 2010. pp. 51–56.

10. McKinney W. Python for data analysis: Data wrangling with Pandas, NumPy, and IPython. " O'Reilly Media, Inc."; 2012.

11. Bernstein FC, Koetzle TF, Williams GJB, Meyer EF, Brice MD, Rodgers JR, et al. The protein data bank. Eur J Biochem. Wiley Online Library; 1977;80: 319–324.

12. Chang A, Scheer M, Grote A, Schomburg I, Schomburg D. BRENDA, AMENDA and FRENDA the enzyme information system: new content and tools in 2009. Nucleic Acids Res. Oxford Univ Press; 2009;37: D588–D592.

13. Bava KA, Gromiha MM, Uedaira H, Kitajima K, Sarai A. ProTherm, version 4.0: thermodynamic database for proteins and mutants. Nucleic Acids Res. Oxford Univ Press; 2004;32: D120–D121.

14. Keseler IM, Collado-Vides J, Gama-Castro S, Ingraham J, Paley S, Paulsen IT, et al. EcoCyc: a comprehensive database resource for Escherichia coli. Nucleic Acids Res. Oxford Univ Press; 2005;33: D334–D337.

15. Hubbard T, Barker D, Birney E, Cameron G, Chen Y, Clark L, et al. The Ensembl genome

database project. Nucleic Acids Res. 2002;30: 38–41.

16. Zhang Y. Progress and challenges in protein structure prediction. Curr Opin Struct Biol. Elsevier; 2008;18: 342–348.

17. Jauch R, Yeo HC, Kolatkar PR, Clarke ND. Assessment of CASP7 structure predictions for template free targets. Proteins: Struct Funct Bioinf. Wiley Online Library; 2007;69: 57–67.

18. Battey JND, Kopp J, Bordoli L, Read RJ, Clarke ND, Schwede T. Automated server predictions in CASP7. Proteins: Struct Funct Bioinf. Wiley Online Library; 2007;69: 68–82.

19. Moult J, Fidelis K, Kryshtafovych A, Rost B, Hubbard T, Tramontano A. Critical assessment of methods of protein structure prediction - Round VII. Proteins: Struct Funct Bioinf. Wiley Online Library; 2007;69: 3–9.

20. Kopp J, Bordoli L, Battey JND, Kiefer F, Schwede T. Assessment of CASP7 predictions for template-based modeling targets. Proteins: Struct Funct Bioinf. Wiley Online Library; 2007;69: 38–56.

21. Martí-Renom MA, Stuart AC, Fiser A, Sánchez R, Melo F, Šali A. Comparative protein structure modeling of genes and genomes. Annu Rev Biophys Biomol Struct. Annual Reviews 4139 El Camino Way, PO Box 10139, Palo Alto, CA 94303-0139, USA; 2000;29: 291–325.

22. Liwo A, Lee J, Ripoll DR, Pillardy J, Scheraga HA. Protein structure prediction by global optimization of a potential energy function. Proceedings of the National Academy of Sciences. National Acad Sciences; 1999;96: 5482–5485.

23. Simons KT, Kooperberg C, Huang E, Baker D. Assembly of protein tertiary structures from fragments with similar local sequences using simulated annealing and Bayesian scoring functions. J Mol Biol. Elsevier; 1997;268: 209–225.

24. Wu S, Skolnick J, Zhang Y. Ab initio modeling of small proteins by iterative TASSER simulations. BMC Biol. BioMed Central Ltd; 2007;5: 17.

25. Das R, Qian B, Raman S, Vernon R, Thompson J, Bradley P, et al. Structure prediction for CASP7 targets using extensive all-atom refinement with Rosetta@ home. Proteins: Struct Funct Bioinf. Wiley Online Library; 2007;69: 118–128.

26. Zhang Y. Template-based modeling and free modeling by I-TASSER in CASP7. Proteins: Struct Funct Bioinf. Wiley Online Library; 2007;69: 108–117.

27. Bowie JU, Luthy R, Eisenberg D. A method to identify protein sequences that fold into a known three-dimensional structure. Science. American Association for the Advancement of Science; 1991;253: 164–170.

28. Jones DT, Taylort WR, Thornton JM. A new approach to protein fold recognition. Nature. Nature Publishing Group; 1992;

29. Cozzetto D, Kryshtafovych A, Fidelis K, Moult J, Rost B, Tramontano A. Evaluation of template-based models in CASP8 with standard measures. Proteins: Struct Funct Bioinf.

Wiley Online Library; 2009;77: 18–28.

30. Chang RL, Andrews K, Kim D, Li Z, Godzik A, Palsson BO. Structural systems biology evaluation of metabolic thermotolerance in Escherichia coli. Science. American Association for the Advancement of Science; 2013;340: 1220–1223.

31. Zhang Y, Thiele I, Weekes D, Li Z, Jaroszewski L, Ginalski K, et al. Three-dimensional structural view of the central metabolic network of Thermotoga maritima. Science. American Association for the Advancement of Science; 2009;325: 1544–1549.

32. Xu D, Zhang Y. Ab Initio structure prediction for Escherichia coli: towards genome-wide protein structure modeling and fold assignment. Sci Rep. Nature Publishing Group; 2013;3.

33. Zhou H, Gao M, Kumar N, Skolnick J. SUNPRO: Structure and function predictions of proteins from representative organisms. 2012; Available: http://cssb.biology.gatech.edu/sites/default/files/sunpro_unpublished.pdf

34. Roy A, Kucukural A, Zhang Y. I-TASSER: a unified platform for automated protein structure and function prediction. Nat Protoc. Nature Publishing Group; 2010;5: 725–738.

35. Bakan A, Meireles LM, Bahar I. ProDy: protein dynamics inferred from theory and experiments. Bioinformatics. Oxford Univ Press; 2011;27: 1575–1577.

36. Jaroszewski L, Pawlowski K, Godzik A. Multiple Model Approach: Exploring the Limits of Comparative Modeling. J Mol Med. Springer-Verlag; 1998;4: 294–309.

37. Laskowski RA, MacArthur MW, Moss DS, Thornton JM. PROCHECK: a program to check the stereochemical quality of protein structures. J Appl Crystallogr. International Union of Crystallography; 1993;26: 283–291.

38. Zhang Y. I-TASSER: Fully automated protein structure prediction in CASP8. Proteins: Struct Funct Bioinf. Wiley Online Library; 2009;77: 100–113.

39. Cock PJA, Antao T, Chang JT, Chapman BA, Cox CJ, Dalke A, et al. Biopython: freely available Python tools for computational molecular biology and bioinformatics. Bioinformatics. Oxford Univ Press; 2009;25: 1422–1423.

40. Rice P, Longden I, Bleasby A. EMBOSS: the European Molecular Biology Open Software Suite. Trends Genet. 2000;16: 276–277.

41. Pruitt KD, Brown GR, Hiatt SM, Thibaud-Nissen F, Astashyn A, Ermolaeva O, et al. RefSeq: an update on mammalian reference sequences. Nucleic Acids Res. 2014;42: D756–63.

42. Zhang Y. I-TASSER server for protein 3D structure prediction. BMC Bioinformatics. 2008;9: 40.

43. Case DA, Babin V, Berryman J, Betz RM, Cai Q, Cerutti DS, et al. Amber 14. University of California; 2014; Available: https://orbilu.uni.lu/handle/10993/16614

44. Yčas M. On earlier states of the biochemical system. J Theor Biol. Elsevier; 1974;44:

145–160.

45. Jensen RA. Enzyme recruitment in evolution of new function. Annual Reviews in Microbiology. Annual Reviews 4139 El Camino Way, PO Box 10139, Palo Alto, CA 94303-0139, USA; 1976;30: 409–425.

46. Horowitz NH. On the evolution of biochemical syntheses. Proc Natl Acad Sci U S A. National Academy of Sciences; 1945;31: 153.

47. Eddy SR. A new generation of homology search tools based on probabilistic inference. Genome Inform. World Scientific; 2009. pp. 205–211.

48. Murphy KP, Freire E. Structural energetics of protein stability and folding cooperativity. J Macromol Sci Part A Pure Appl Chem. 1993;65: 1939–1946.

49. Oobatake M, Ooi T. Hydration and heat stability effects on protein unfolding. Prog Biophys Mol Biol. 1993;59: 237–284.

50. Ku T, Lu P, Chan C, Wang T, Lai S, Lyu P, et al. Predicting melting temperature directly from protein sequences. Comput Biol Chem. Elsevier; 2009;33: 445–450.

51. Dill KA, Ghosh K, Schmit JD. Physical limits of cells and proteomes. Proc Natl Acad Sci U S A. 2011;108: 17876–17882.

52. Saito M, Takemura N, Shirai T. Classification of ligand molecules in PDB with fast heuristic graph match algorithm COMPLIG. J Mol Biol. 2012;424: 379–390.

53. O'Brien EJ, Lerman JA, Chang RL, Hyduke DR, Palsson BØ. Genome-scale models of metabolism and gene expression extend and refine growth phenotype prediction. Mol Syst Biol. Wiley Online Library; 2013;9.

54. Chang RL, Xie L, Bourne PE, Palsson BO. Antibacterial mechanisms identified through structural systems pharmacology. BMC Syst Biol. BioMed Central Ltd; 2013;7: 102.

55. Aziz RK, Bartels D, Best AA, DeJongh M, Disz T, Edwards RA, et al. The RAST Server: rapid annotations using subsystems technology. BMC Genomics. 2008;9: 75.

56. Ye Y, Godzik A. Flexible structure alignment by chaining aligned fragment pairs allowing twists. Bioinformatics. 2003;19 Suppl 2: ii246–55.

57. Pedregosa F, Varoquaux G, Gramfort A, Michel V, Thirion B, Grisel O, et al. Scikit-learn: Machine Learning in Python. arXiv [cs.LG]. 2012.

58. Jones E, Oliphant T, Peterson P, Others. SciPy: Open source scientific tools for Python, 2001--. URL http://www scipy org. 2007;73: 86.
